# Composite Membranes for High Temperature PEM Fuel Cells and Electrolysers: A Critical Review

**DOI:** 10.3390/membranes9070083

**Published:** 2019-07-11

**Authors:** Xinwei Sun, Stian Christopher Simonsen, Truls Norby, Athanasios Chatzitakis

**Affiliations:** Centre for Materials Science and Nanotechnology, Department of Chemistry, University of Oslo, FERMiO, Gaustadalléen 21, NO-0349 Oslo, Norway

**Keywords:** proton exchange membrane, composite membrane, inorganic fillers, proton conductivity, mixed proton electron conducting membranes, positrode, negatrode, fuel cells, electrolysers

## Abstract

Polymer electrolyte membrane (PEM) fuel cells and electrolysers offer efficient use and production of hydrogen for emission-free transport and sustainable energy systems. Perfluorosulfonic acid (PFSA) membranes like Nafion^®^ and Aquivion^®^ are the state-of-the-art PEMs, but there is a need to increase the operating temperature to improve mass transport, avoid catalyst poisoning and electrode flooding, increase efficiency, and reduce the cost and complexity of the system. However, PSFAs-based membranes exhibit lower mechanical and chemical stability, as well as proton conductivity at lower relative humidities and temperatures above 80 °C. One approach to sustain performance is to introduce inorganic fillers and improve water retention due to their hydrophilicity. Alternatively, polymers where protons are not conducted as hydrated H_3_O^+^ ions through liquid-like water channels as in the PSFAs, but as free protons (H^+^) via Brønsted acid sites on the polymer backbone, can be developed. Polybenzimidazole (PBI) and sulfonated polyetheretherketone (SPEEK) are such materials, but need considerable acid doping. Different composites are being investigated to solve some of the accompanying problems and reach sufficient conductivities. Herein, we critically discuss a few representative investigations of composite PEMs and evaluate their significance. Moreover, we present advances in introducing electronic conductivity in the polymer binder in the catalyst layers.

## 1. Introduction

Molecular hydrogen, or hydrogen gas is one of our most important chemicals, and the technologies to produce, transport, store, and use it are mature and safe [1]. Today, it is mainly produced with little energy cost, but large CO_2_ emissions from fossil resources. As the world must move towards renewable energy, electrolysis (in addition to use of biomass) will become of increasing importance, and it is imperative to optimise performance and reduce the cost and environmental impact (e.g., use of rare elements) of electrolysis [2,3,4]. Alkaline (KOH-based) electrolysis (AEs) is the leading industrial technology [5], but solid-electrolyte electrolysers, notably polymer electrolyte membrane electrolysers (PEMEs), are catching up, offering many advantages. For the use of hydrogen gas as intermittent storage of electricity and in transport, the fuel cell is an essential component, and while phosphoric acid and alkaline electrolysers have been leading industrially, solid-electrolyte fuel cells are considered safer and more efficient. In particular, polymer electrolyte membrane fuel cells (PEMFCs) are becoming industry leaders for hydrogen-driven fuel cell electrical vehicles (FCEVs; cars and trucks), trains and ships, and small- and intermediate-sized autonomous and grid-balancing energy systems [6,7,8].

**Figure 1:**
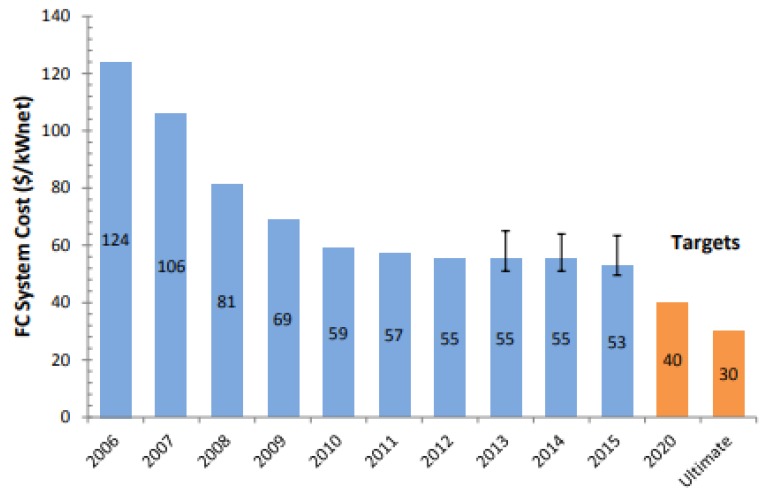
Cost-analysis of an 80 kW_net_ automotive PEMFC-based on projection to a volume of 500,000 units/year [10].

The advantages of using a stationary solid polymer electrolyte comprise high power density, rapid start-up, and system simplicity. Nevertheless, durability and cost remain primary challenges for PEMFCs to become commercially competitive with conventional vehicle technologies. According to a cost-analysis from 2015, the cost of an 80/kW_net_ automotive PEMFC operated on direct hydrogen gas is projected to be $53/kW_net_ when manufactured at a volume of 500,000 units/year (Figure 1), whereas the cost of the membrane accounts for ca. 5.6% ($2.95/kW) [9]. The target cost of automotive PEMFC systems for 2020 based on current technology is approximately $40/kW_net_ [10]. This implies a reduction in the cost of the membrane by $0.8/kW. Further system cost reduction to $30/kW_net_ must be achieved for long-term competitiveness with the current technologies, which corresponds to $1.44/kW cost reduction for the membrane for a market with high volume production. More specifically, PEM materials must possess the required characteristics as listed in Table 1.

The core of traditional PEM electrochemical cells (PEMECs)—treated in many articles and reviews, e.g., Refs. [14,15]—is the membrane electrode assembly (MEA) consisting of a solid-state proton-conductive polymer electrolyte sandwiched between two porous, electronically-conductive and catalytically-active electrodes. The solid proton-conductive electrolyte ensures the conduction of protonic charge carriers (hydrated H_3_O^+^ ions) between the electrodes, and is electronically insulating.

**Figure 2:**
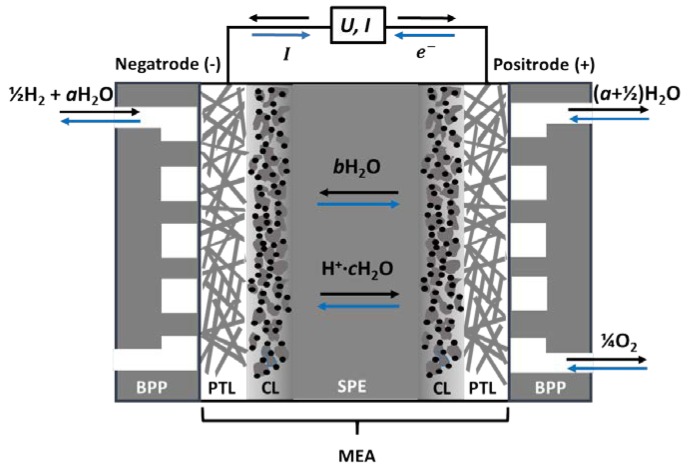
Schematic of PEM electrochemical cells (PEMECs)—fuel cells (PEMFC, black upper arrows) and electrolysers (PEMEs, blue lower arrows). Protons hydrated with *c* bonded and electro-osmotically dragged H_2_O molecules migrate through the solid polymer electrolyte (SPE), the water supplied by *b* back-diffusing water, and a water supplied in a feed gas, so that *a* + *b* = *c*. BPP: bipolar plate gas manifold, PTL: Porous transport layer, CL: catalyst layer, SPE: solid polymer electrolyte, MEA: membrane electrode assembly.

One electrode operates under reducing conditions, exposed to hydrogen gas and water vapour. It is always negative, whether run as an anode in the fuel cell or a cathode in an electrolyser, and we will here refer to it as a negatrode. Similarly, the other electrode is always positive, operating under oxidising conditions in oxygen and water vapour as a cathode in the fuel cell or anode in the electrolyser, and we will here refer to it as a positrode, see Figure 2.

Each of the electrodes comprises a catalyst layer (CL), where the electrocatalysts are dispersed on a nanoporous support to promote charge transfer kinetics by lowering the activation energy. Next comes the more openly porous transport layer (PTL), also acting as a current collector alone or by the help of additional metallic meshes or sinters. The MEA is encased by gas manifold bipolar plates (BPPs) on each side, which direct and distribute gases in flow channels and connect the positrode electronically to the negatrode of the adjacent cell in the case of a PEMEC stack [16].

When PEMECs are operated in fuel cell (PEMFC) mode, humidified hydrogen gas is supplied to the negatrode, where it oxidises to protons and electrons, see Figure 2. The protons migrate to the positrode through the electrolyte and react with oxygen to produce water vapour, while the electrons travel through the external circuit and deliver electrical work. In electrolyser (PEME) mode, the current and all processes are reversed.

It is common to consider the transport of single free protons and to write the negatrode, positrode, and overall reactions forward (fuel cell mode) and backward (electrolyser mode) as:

(1) $$\mathrm{Negatrode}:\;\frac{1}{2}H_{2}\;⇌H^{+}+e^{-}\;|2$$

(2) $$\mathrm{Positrode}:{\;H}^{+}+e^{-}+\frac{1}{4}O_{2}⇌\;\frac{1}{2}H_{2}O\;\;|2$$

(3) $$\mathrm{Overall}:{\;H}_{2}+\frac{1}{2}O_{2}⇌H_{2}O$$

In reality, however, the protonic transport in traditional PEMs takes place by hydrated protons, namely H_3_O^+^ ions solvated by a number *c* of additional H_2_O molecules, which are pulled along in an electroosmotic drag, so that one may consider the charge carrier as H^+^·*c*H_2_O, and write the reactions more generally like

(4) $$\mathrm{Negatrode}:\frac{1}{2}H_{2}+{\mathrm{cH}}_{2}O\;⇌{\;H}^{+}\cdot cH_{2}O+e^{-}\;|2$$

(5) $$\mathrm{Positrode}:{\;H}^{+}\cdot cH_{2}O+e^{-}+\frac{1}{4}O_{2}\;⇌\;\left(c+\frac{1}{2}\right)H_{2}O\;|2$$

(6) $$\mathrm{Overall}:{\;H}_{2}+\frac{1}{2}O_{2}⇌H_{2}O$$

The case of *c* = 0 represents the simplified case from above and also free proton transport in high-temperature water-free proton conductors, while increasing *c* describes systems at lower temperatures, higher relative humidities, and higher contents of adsorbed water or liquid-like condensed water. High *c* is also accompanied with high mobility of protonic charge carriers in the liquid-like water.

As schematically illustrated in Figure 2, the dragged water may partly back-diffuse in the direction opposite to the protonic current, and partly be supplied to the reactant gas and be recirculated from the outlet. On the other hand, as the membrane becomes dehydrated by the electro-osmotic drag, the pores shrink. It is then possible that the back diffusion of water is not enough to avoid dehydration of the membrane, leading to a decrease of efficiency of the fuel cell [17].

Ionic transport in PEMs is a complex matter, with several types of mobile protonic species and pathways. The two classifications used above are the Grotthuss (free proton) and vehicle mechanisms, while surface (or interface) transport is sometimes mentioned as a third type [15,18]. In the Grotthuss mechanism, a proton jumps from one anion, normally the oxide ion in a solvated H_3_O^+^ or stationary -OH^−^, to another. The vehicle mechanism, on the other hand, comprises transport of H_3_O^+^ ions solvated in liquid-like aqueous media. Generally, the breaking and making of bonds in the Grotthuss mechanism involves a higher activation energy than the fluidic diffusion of vehicular species. Moreover, the amount of hydration—the volume of the water phase—decreases with lower relative humidity, usually a result of increasing temperature. Hence, all in all, Grotthuss-type free proton transport tends to become more dominating at higher temperatures and lower relative humidities, while vehicle transport dominates at low temperatures and higher relative humidities. In a fully-hydrated polymer, or at constant relative humidity, the temperature dependencies may appear different, as the state (viscosity) of the water phase changes, while its volume may remain constant. Surface and interface transport of protonic species takes place by protons jumping between neighbouring acidic donor/acceptor sites on the polymer backbone, facing gas or water, respectively, in dry or hydrated membranes, and does as such represent Grotthuss type transport. On the basis of the above, the total protonic conductivity of a polymer membrane depends on its backbone morphology and dynamics, the concentration and acidity of proton donor/acceptor sites, and the resulting hydrophilicity and water content. We may translate this into volume of the conducting phase and concentration and charge mobility of protonic species. For a given polymer, the conductivity becomes a complex function of temperature and water activity (partial pressure) or relative humidity.

Traditional PEMECs are based on perfluorinated polyethylene polymer membranes, which are grafted (branched), sulfonated with concentrated sulphuric acid, neutralised with an alkali such as NaOH, and proton exchanged (hence the use of the name proton exchange membrane (PEM)) to replace Na^+^ with H^+^ (or H_3_O^+^). In contact with water, they swell and form hydrophilic water-filled proton-conducting channels and hydrophobic backbones. They operate typically at 80 °C at high relative humidity (RH, >40%), and cannot withstand much increased temperatures as they dehydrate, causing a drop in proton conductivity, and eventually degrade irreversibly [19].

**Figure 3:**
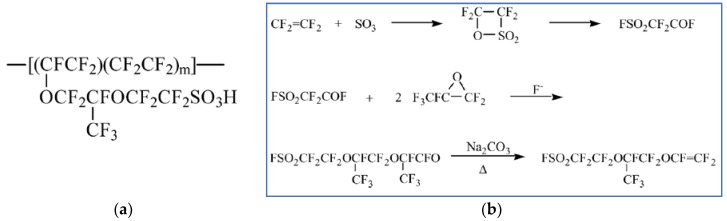
The chemical structure of Nafion (**a**) and the synthesis process for the vinyl ether monomers, the starting point for Nafion (**b**). (**b**) is reprinted with permission from Ref. [20]. Copyright 2004 Elsevier.

Nafion^®^ developed by DuPont in the late 60s is still the state-of-the-art PEM. Initially, Nafion was developed for the chloralkali electrolysers as a permselective separator, but Nafion had oxidative stability, and after proton exchange, also the proton conductivity required for PEMFCs [14,20]. Nafion is composed of a hydrophobic tetrafluoroethylene (TFE) backbone sequence together with a co-monomer that contains pendant side chains of perfluorinated vinyl ethers, which are terminated by perfluorosulfonic acid groups (Figure 3a). The synthetic route of TFE-carrying branches of pendant sulfonic acid groups is given in Figure 3b.

The polytetrafluoroethylene (PTFE) hydrophobic matrix contains well-connected hydrophilic ionic clusters that despite their low ion exchange capacity (IEC) show high proton conductivity below 90 °C. Moreover, this unique fluorocarbon polymer structure is responsible for the good mechanical and chemical stability [21,22].

The performance of low-temperature PEMFCs (LT-PEMFC ≤ 80 °C) is in general limited by fuel crossover, CO poisoning on the anode Pt catalyst, slow electrode reaction kinetics, complex water management, inefficient cooling heat exchange, and little usage of waste heat [23,24]. As mentioned, Nafion as the state-of-the-art LT-PEM material relies on a high level of hydration in order to accommodate the proton transport and reach sufficient proton conductivity (100 mS/cm) at temperatures up to 80 °C. At higher temperatures, the chemical and mechanical stability of Nafion is compromised because of the low glass transition temperatures of the perfluoroaliphatic polymer chains of PFSAs. Moreover, at these temperatures, the ionic clusters dehydrate and the protonic conductivity is heavily reduced, leading to a significant decrease in the PEMFC performance. Another concern is the high cost of Nafion, which is holding back mass production and full commercialization [25,26]. Ideally, the operating temperature of a PEMFC should be above 100 °C, and this has stimulated efforts to develop proton conductors for higher temperature operation in the last two decades. However, also PEM electrolysers would benefit from higher operating temperatures for many of the same reasons. In addition, one may supply waste heat or steam and increase the electrical efficiency by operating at high temperatures (e.g., above 100 °C).

**Figure 4:**
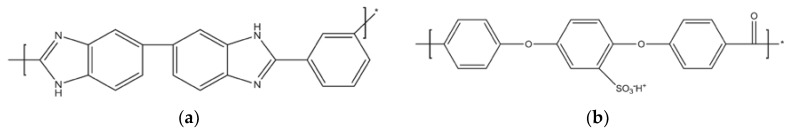
Structures of PBI (**a**) and SPEEK (**b**).

Strategies to increase the operating temperatures of PEMs involve the use of heterocyclic polymers like the thermoplastic polybenzimidazole (PBI); its structure can be seen in Figure 4a.

The original idea is that the N atoms of PBI will bond protons weaker than fully covalent carbon or oxide ions would in other polymers and hence act as suitable proton donors and acceptors for free proton transport at elevated temperatures. However, acid-doping has turned out necessary to achieve considerable proton conductivity in PBI.

Another route has been the high-temperature thermoplastic polyether-ether-ketone (PEEK), where again acid-doping in order to form sulfonated PEEK (SPEEK) is necessary to achieve appreciable proton conductivity (Figure 4b) [27]. The mobility of protons from or in the acid increases with temperature, and generally one needs well above 120 °C, typically 160 °C, to yield a sufficient proton conductivity. However, at these temperatures, the long term stability of these polymers is compromised and the acid doping can sip out and corrode metal interconnections [13].

**Figure 5:**
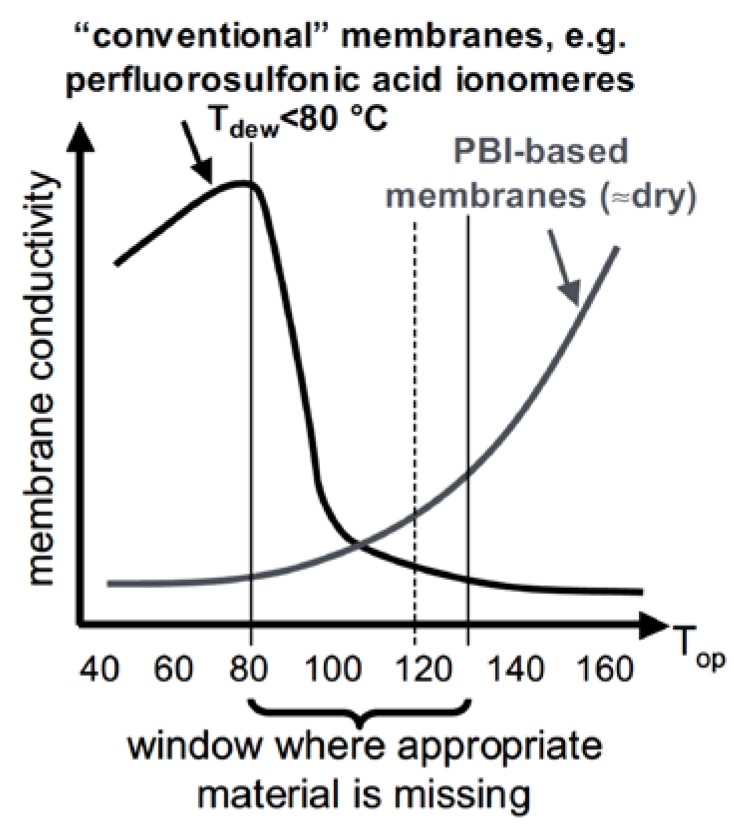
Membrane conductivity based on perfluorosulfonic acid (PFSA) ionomers and PBI, displaying the conductivity gap from 80 °C to 130 °C. Reprinted with permission from Ref. [28]. Copyright John Wiley & Sons, Ltd.

As nicely depicted by Wieser (Figure 5), a “conductivity-gap” exists at intermediate temperatures and especially around 120 °C which is the target temperature as given in Table 1 [28].

A number of studies attempt to improve the conductivity and stability at temperatures in the conductivity-gap by dispersing a secondary ceramic phase (filler) so as to make a polymer-ceramic (pemcer) composite. Ceramics are added to both LT polymers like Nafion, as well as to PBI and SPEEK. The fillers are intended to increase the water retention due to their hygroscopicity, reduce fuel and oxygen cross-over, induce fast proton mobility at the interfaces, scavenge harmful radicals, and finally, improve the mechanical properties [29]. Here, we review these materials, their principles of operation and their performances. It is appropriate to ask how sound the principles are and if the materials work as claimed.

For instance, hygroscopic ceramic fillers may “save” the polymer by retaining water during a critical overheating, but one rarely finds a well-founded thermodynamic or physicochemical argument for the effect of the ceramic. The use of inert particles in order to avoid gas crossover can eventually impede proton transport, and one might just as well increase the thickness of the membrane. Fast transport in polymer–ceramic interfaces is possible, but to beat the highly conducting liquid aqueous phase is hard, and little is put forward e.g., in terms of charge separation or space charge effects to rationalise why and how it would work. Scavenging harmful oxidising radicals like OH* or catalyst poisons like CO will be highly beneficial, but could possibly be applied better in the electrode matrix than in the membrane, although Macauley et al. [30] recently showed that a CeO_2_-scavenger-modified PFSA has the potential to achieve a 25,000 h of heavy-duty fuel cell durability. Ceramic dispersions may increase the hardness and temporarily the thermal stability of the membrane, but they also increase the brittleness; hence, we may ask if they are really of help, or whether the toughness of the pure polymer is a better choice. A few important studies highlighting several approaches to increase the mechanical and chemical properties of PEMs can be found in Refs. [31,32].

The next section reviews and discusses composite membranes based on PFSAs with Nafion as the prominent example, while the following two sections cover composite membranes based on PBI and SPEEK. After that, we introduce briefly the progress on mixed electron–proton-conducting polymers, which are particularly interesting for the efficient utilization of the electrocatalysts in the CL. By the end of this review, we hope to advocate and foster deeper physicochemical analysis for better founded strategies on how composites may help develop high-temperature proton-conducting polymer electrolyte membranes.

## 2. Long Side Chain PFSA Polymer: Nafion-Based Composite Membranes

Protonic conductivity of Nafion membranes depends heavily on the degree of hydration and the availability of the sulfonic acid sites, which attract water and form solvated hydronium ions (H_3_O^+^) as the protonic transport vehicle. The conductivity of fully-humidified Nafion reaches 0.12 S/cm at 80 °C and atmospheric pressure, and decreases by several orders of magnitude with decreasing relative humidity [33,34]. As mentioned earlier, one common approach to alleviate membrane dehydration at elevated temperatures is by introducing ceramic fillers. Possible mechanisms are still under debate, but there are indications that the improved proton conduction is due to the water retention properties resulting from an increased tortuosity induced by the fillers inside the membrane, and enhanced crystallinity especially for elevated pressure operation [35,36]. In the following section, we review in more detail some promising Nafion-based composite membranes for high-temperature fuel cells or electrolysers.

### 2.1. Hydrophilic Inorganic Material

Dispersed “hygroscopic” oxides, such as SiO_2_ [37,38], TiO_2_ [39,40], ZrO_2_ [41,42] and Al_2_O_3_ [36], have been reported to form dynamic cross-links with sulfonic acid groups of Nafion, thereby increasing the porosity and improving water retention. They also decrease the gas crossover with respect to bare Nafion. Minimum cell resistance is achieved at around 140 °C, where physisorbed water is reported to be desorbed from the investigated inorganic fillers [36]. Up to 10 wt.% of the oxide can be added to Nafion without a significant decrease in the protonic conductivity [43]. Even better protonic conductivity can be achieved by sulfonating the oxides [44,45,46].

**Figure 6:**
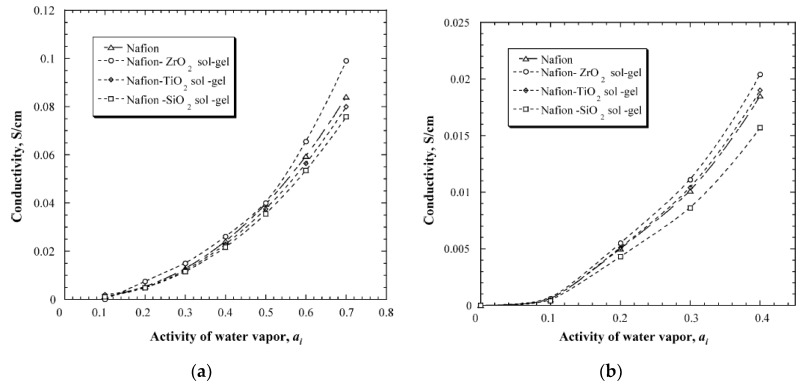
The conductivity of the measured membranes at different water activities at 90 °C (**a**) and 120 °C (**b**). Reprinted with permission from Ref. [47]. Copyright 2005 Elsevier.

An in-situ sol-gel process was used to make Nafion membranes containing ZrO_2_, SiO_2_ and TiO_2_. This process used pre-cast membranes that were cleaned in hydrogen peroxide solution and underwent an ion exchange from H^+^ to Na^+^ by reacting the membrane with NaOH solution. The membranes were then heat-treated in a vacuum furnace before being immersed in a 90% ethanol-solution to hydrate and swell the membrane. The swelled membrane was then placed in the metal-precursor solution that decomposed to the metal oxide when reacted with water. The idea behind this method is that the membranes will serve as the template that directs the morphology, particle size and growth rate of the metal oxide [47]. Results from this study showed that the composite membrane either retained or increased the water uptake. The membranes with ZrO_2_ increased the water retention capacity by 33% and 45% at 90 °C and 120 °C, respectively, TiO_2_ by 20–25%, and SiO_2_ had a 15% increase at 120 °C. The conductivity measurements, however, showed that neither TiO_2_ nor SiO_2_ gave any increase in conductivity compared to the recast Nafion, and the ZrO_2_ had an increase of merely 8–10% compared to pure Nafion (Figure 6). The authors concluded that the increase in water uptake does not necessarily result in a higher conductivity [47].

**Figure 7:**
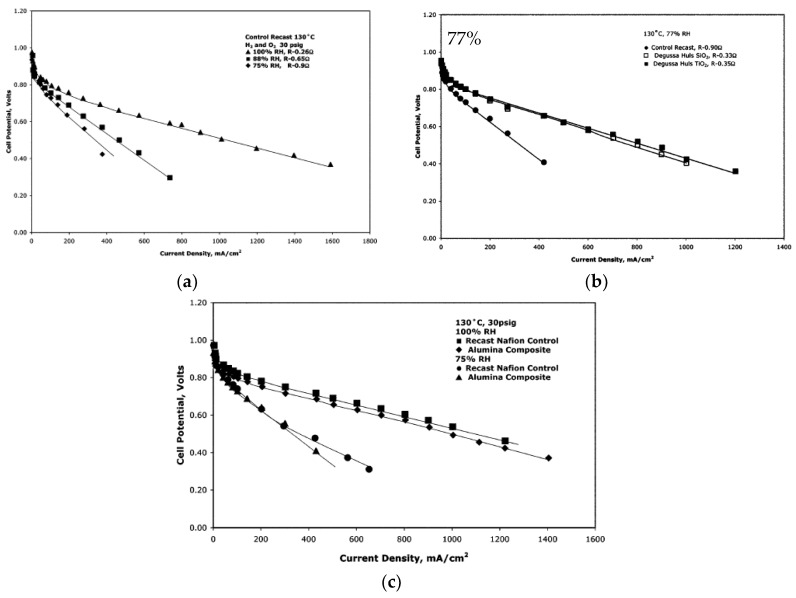
The current density plot for recast Nafion at 130 °C, 30 psig and using H_2_/O_2_ as reagent gases (**a**). The current density plots for membranes containing SiO_2_ and TiO_2_ at 130 °C at 77% RH using a pressure of 30 psig and H_2_/O_2_ as reagent gases (**b**). The current density plot for Nafion membrane and an Al_2_O_3_ composite membrane at 130 °C, 30 psig and using H_2_/O_2_ as reagent gases (**c**). Reprinted with permission from Ref. [24]. Copyright 2006 American Chemistry Society.

Another synthesis method comprised pre-made nanoparticles of SiO_2_, Al_2_O_3_, TiO_2_ and ZrO_2_. These particles were mixed in a 5% Nafion solution mixed with double its volume of isopropyl acid. The total mass of the inorganic particles was 3 wt.%, and the membranes were standardized to a thickness of 125 μm [24]. The membranes were tested at 130 °C at relative humilities between 100% and 75% (Figure 7). A 68% reduction of resistance was observed for the membrane with SiO_2_ particle sizes of 0.2–0.3 µm and a surface area of 90 g/m^2^, compared to the plain Nafion at 75% RH. The closer to 100% RH, the smaller the differences in proton transport resistances. For the TiO_2,_ the same trend was observed with a smaller difference in resistance to the higher the relative humidity, and at 75% RH there was a 61% decrease in the resistance. The particle size in this membrane was 1–2 µm. For the alumina composite (Figure 7c), the membrane with particle size of 25 µm showed better results than 1 µm, where the resistance at 75% RH decreased by 22%, but at 100% RH the composite membrane showed a higher resistance than the plain Nafion. The membrane with 6 µm ZrO_2_ particles showed a decrease of 40% in resistance at 75% RH compared to the recast Nafion membrane [24].

**Figure 8:**
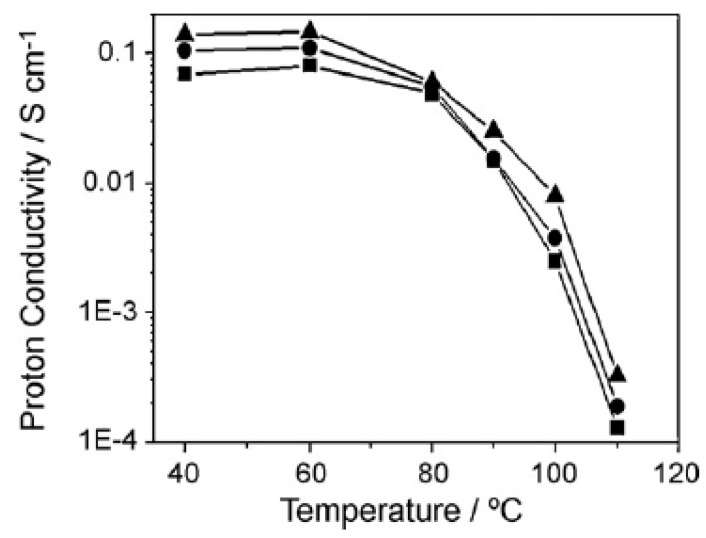
The proton conductivity of membranes without external humidification. Triangle is zirconia-doped, circle is silica-doped membrane and square is recast Nafion. Reprinted with permission from Ref. [48]. Copyright 2010 Elsevier.

Another study used a self-assembly process by mixing a Nafion-solution with M-methyl-2-pyrrolidone and all other solvents were removed by heat treatment. The mixing in the metal precursor solution and the subsequent hydrolysing reaction produced metal oxide nanoparticles that are stabilized by the Nafion matrix. The solution was then heat treated to produce the final membrane [48]. This study used composite membranes made with zirconia and silica particles, and based on the water uptake measurements, the composite membranes showed a higher water content at 100 °C, where below 20% RH the improvement is minimal. At higher than 40% RH levels, a water uptake between 2 to 3 times higher for the composite membranes compared to plain Nafion was observed. The conductivity of the membranes was tested without external humidification in order to assess the water retention capacity of the membranes. The results indicated that the zirconia-doped membrane had the best conductivity that can come from the water retention compared to the Nafion, and the conductivity at 100 °C was six times higher, reaching ca. 0.01 S/cm (Figure 8). The silica-doped membrane also showed a higher conductivity but less than the zirconia-doped membrane [48].

**Figure 9:**
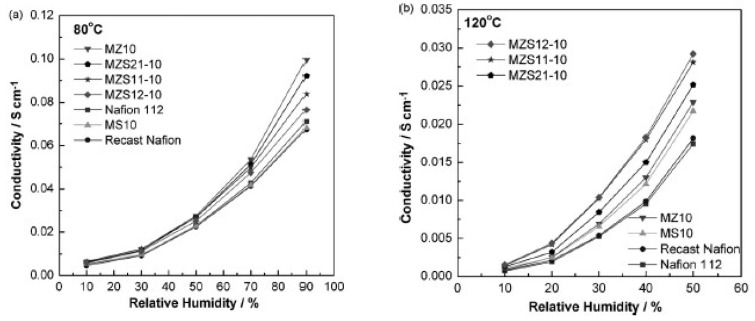
The measured conductivity of the membranes over different RH levels, where the membranes are labeled MZSxy-10 and x:y is Zr:Si, at 80 °C (**a**) and at 120 °C (**b**). Reprinted with permission from Ref. [49]. Copyright 2008 Elsevier.

In a similar work, silica, zirconia and their combinations were used as inorganic “dopants” to Nafion membranes. The membranes were made by mixing premade surfactant-free particles into a Nafion solution and then heat treated. The membranes were made with 10 wt.% inorganic fillers [49]. The results from this study showed that the silica-doped membrane had a lower water uptake than the recast Nafion and the commercial Nafion 112. The highest water uptake was in the zirconia-modified membrane, where the water uptake increased merely from 38.3% to 39%. All the other combinations of the binary oxide particles followed a linear trend depending on the zirconia concentration. The conductivity of the membranes at 80 °C showed that the zirconia-modified membrane had the highest conductivity of 0.1 S/cm at 90% RH. The recast Nafion, commercial Nafion 112 and the silica-doped membrane, all had a conductivity of 0.07 S/cm (Figure 9a). At 120 °C and 50% RH, the recast Nafion and commercial Nafion 112 had a conductivity of 0.0175 S/cm, while the membrane with two parts silica and one part zirconia as the inorganic dopant had a conductivity of ca. 0.03 S/cm (Figure 9b). This is a slight improvement from the pure silica and zirconia-doped membranes, that showed conductivity values of 0.024 and 0.026 S/cm, respectively [49].

Chalkova et al. used titania powders with particle sizes of 0.1–1 µm and 0.2–0.3 µm, which were mixed into 5% Nafion solution and heat treated to obtain 10 wt.% TiO_2_ Nafion membranes with a thickness of 80 µm [50]. The current density of the membranes with titania showed a significant improvement compared with the recast Nafion membrane over all RH values, at temperatures of 80 °C and 120 °C. Comparing the composite membranes at a cell voltage of 0.6 V, the one with particles size of 0.2—0.3 µm performed 1.2 to 1.7 times better at 26% and 50% RH at 80 °C and 1.4 to 4 times better at 26% and 50% RH compared to the membrane with a particle size of 0.1–1 µm. It is also noted that the membrane with particles size of 0.2–0.3 µm was less affected by the change in relative humidity. These results suggest that the surface properties of TiO_2_ somehow can decrease the resistance of the proton diffusion. The surface properties are dependent on the morphology, surface area, and the electric double layer created by the charged surface [50].

Finally, the outstanding mechanical properties of carbon nanotubes (CNTs) make them an interesting filler material for composite reinforcement [51]. Nafion filled with sulfonic acid functionalized single-walled carbon nanotubes (S-SWCNTs) exhibits almost one order of magnitude higher ionic conductivity than that of Nafion1135 even above 100 °C, as well as enhanced mechanical stability [52]. The composite membrane gives a maximum power density of 260 mW/cm^2^ at 0.42 V, whereas the Nafion 1135 membrane gives 210 mW/cm^2^ at 0.39 V.

### 2.2. Metal Organic Frameworks (MOFs)

Another category of inorganic fillers recently reported to increase the conductivity of Nafion for operation at low RH is metal organic frameworks (MOFs). Some MOFs become proton conducting after the incorporation of protonic charge carriers, such as water, acids and heterocycles into their pores [53]. In general, increased proton conductivity can be achieved from polymer/MOF composites through two ways, either drenching the pores of MOFs with proton carriers (e.g., phytic@MIL [54], PIL@MIL [55], acids@MIL-101 [56], ZIF-8 and ZIF-67 [57], sulfonated MIL [58], ZIF-8/CNT network [59], Fe-MIL-101-NH_2_ [60]), or through modification of their organic ligand with functional groups (-SO_3_H, -NH_2_) to increase the acidity and hydrophilicity (e.g., sulfonated Zr-MOF-808 [61], S-UiO-66@GO [62]). Nevertheless, very few of them have actually been tested for PEMFC operation [56,63,64]. In this chapter, we will briefly touch upon a few recent developments reported in the literature regarding fillers based on MOFs.

Sadakiyo et al. controlled the hydrophilicity of a class of MOF materials based on (NR_3_(CH_2_COOH))(MCr(ox)_3_)·*n*H_2_O, where R = methyl, ethyl or n-butyl and M = Mn or Fe [65]. The MOFs are simply named as R-MCr, and the most hydrophilic sample was the Me-FeCr, which at room temperature showed a proton conductivity of 0.1 mS/cm at 65% RH. The performance and the effect of this MOF have not been investigated in a mixed conducting membrane or at higher temperatures and even lower RH levels.

Li et al. immobilized phytic acid, which can be obtained from plants and contains a high concentration of phosphate groups, onto MIL101 via vacuum-assisted impregnation [54]. Composite Nafion/phytic@MIL101 membranes were synthesized by the solution casting method with varying amounts of MIL101 and phytic@MIL101. The proton conductivities were measured at 80 °C in different RH levels, showing conductivities of the best performing composite (which had 12 wt.% of phytic@MIL101) of 61 mS/cm and 0.7 mS/cm at 57% and 11% RH, values that are 2.8 and 11 times higher than pure Nafion. The improved proton conductivity is assigned to the continuous channels formed by the MIL101 and the phosphate groups available from the phytic acid. It is worth mentioning that the authors measured the conductivity of the pure Nafion at 80 °C and 100% RH, and it was found to be of the order of 100 mS/cm, giving confidence in their methodology. On the other hand, in their schematic representation of the mechanism for the improved proton conduction, the MOFs appear to be smaller than the Nafion channels. This is questionable, as the MOF appears to be hundreds-of-nanometers-big particles. It is not clear if a percolating network is formed between the modified MOFs, and in fact the Nafion/MIL101 composite performed worse than the pure Nafion. So, is it possible that phosphate groups are leaking from the phytic acid “dope” Nafion? The leakage test was performed in water, but can it accurately simulate the conditions in Nafion?

A high-performing Nafion/MOF composite membrane was developed by Yang et al. [66]. In this work, the porous framework ZIF-8 is grown on graphene oxide (GO) and the ZIF-8@GO composite is introduced into Nafion. The authors reported a proton conductivity of 280 mS/cm at 120 °C, at 40% RH, a performance that is 55 times higher than that of Nafion (5 mS/cm). This high performance is attributed to the high water retention capability of the composite, as well as to the unique monolayer structure of ZIF-8@GO. Unfortunately, the authors did not test this membrane in an actual PEMFC or PEMWE in order to verify the positive effects of such a composite membrane under realistic conditions.

One of the first studies of a Nafion/MOF composite membrane employed in a PEMFC is described by Tsai et al. [67]. Herein, the authors mixed MOFs with 1D microporous channels (CPO-27(Mg) and MIL-53(Al)) with Nafion through a simple solution recast protocol. The filler content was kept at 3 wt.% as this was the maximum amount of filler that a homogeneous membrane could obtain. The composite with CPO-27(Mg) had the higher water uptake and protonic conductivity, which was measured at 50 °C and 100% RH. The PEMFC performance of the composite membranes against the pure Nafion one was compared at 100% RH and at different operating temperatures. The Nafion/CPO-27(Mg) composite showed the highest power densities across all temperatures and for example at 50 °C the performance was 74% higher than that of Nafion. Unfortunately, the authors tested only this composite at lower RH, 15%, therefore a comparison with the other membranes cannot be made for low RH values. Nevertheless, the Nafion/CPO-27(Mg) showed exceptional performance at low RH values compared to the high one, and at some temperatures, the power densities were even higher, e.g., at 50 °C and 15% RH, the power density was 853 mW/cm^2^ and 818 mW/cm^2^ at 100% RH. The performance of such a composite should also be tested at high temperatures and both low and high RH.

**Figure 10:**
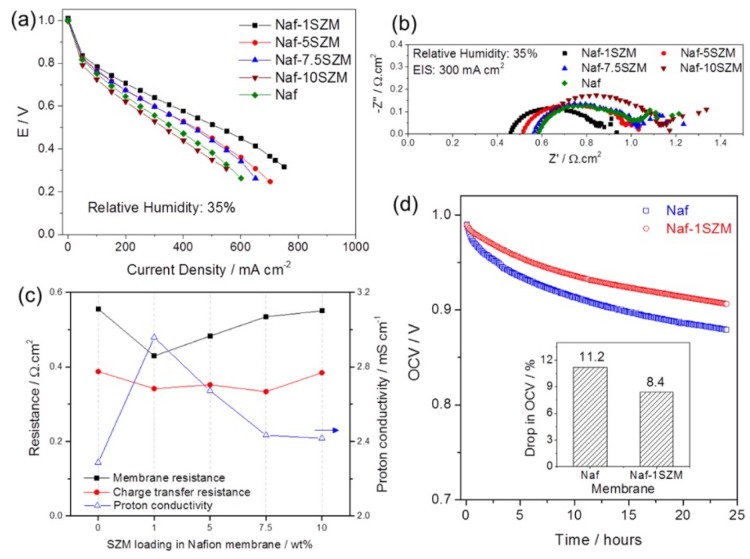
Polarization curves (**a**). Nyquist plots obtained with EIS (**b**), membrane resistances, proton conductivities and charge transfer resistances as a function of the SZM content in the composite (**c**). Stability runs as obtained for 24 h at 35% RH, at 80 °C (**d**). The inset shows the OCP drop in percentage (%). Reprinted with permission from Ref. [61] under a Creative Commons Attribution (CC-BY) License. Copyright 2016 American Chemical Society.

Recently, Rao et al. incorporated the UiO-66-NH_2_ tethered on GO into Nafion [68]. The composite membrane operated at 90 °C both in 95% RH and reportedly under anhydrous conditions with conductivities of 303 and 3.4 mS/cm, respectively. These values were approx. 1.57 and 1.88 times higher than the conductivities measured for recast Nafion, assigned to a synergy between the vehicle and Grotthuss mechanisms. Another UiO-66-based MOF employed in composite Nafion membranes was presented by Donnadio et al. [69]. In this work, a set of composites with the UiO MOF that was either sulfonated or not was tested under different temperatures and RH. The results showed a slight improvement of the proton conductivity at 110 °C; at 50% RH for the composite membranes, either the MOF was sulfonated or not. The optimum filler content was 2 wt.% and independent of the presence of sulfonic groups. It was speculated that the improvement is due to the filler modifying the ionomer’s structural features. The authors conclude and imply that sulfonation of this particular MOF does not improve the performance, and other functional groups, such as phosphates, are suggested for further research. Patel et al. synthesized a composite membrane based on Nafion and superacid sulfonated Zr-MOF (SZM) [61]. The water uptake of the composite membrane was constantly higher at different RH compared to the pure Nafion ones. Therefore, the performance of a PEMFC operated at 80 °C and 35% RH was also improved, showing also increased proton conductivity, as seen from EIS measurements (Figure 10).

The composite with 1 wt.% SZM improved by 34% the voltage of the PEMFC at 0.5 A/cm^2^, while the proton conductivity was increased by 23%.

### 2.3. Solid Acids

The materials referred to in the following, classified as solid acids in the literature and therefore used as fillers for polymer membranes, are in fact not bulk proton conductors, but mainly conduct protons in adsorbed water. This is worth taking into account when their potential and reported effects on hydration and proton conductivity of composites are evaluated.

Shao et al. compared the performance of a composite Nafion membrane with phosphotungstic acid (H_3_PW_12_O_40_·*n*H_2_O, PWA) supported on silica gel against Nafion 115 [70]. It was found that the Nafion\SiO_2_\PWA had the highest water uptake, as well as the highest proton conductivity at 100 °C under a wide range of RH values. The authors assembled a single PEMFC, which was operated at 110 °C and 70% RH. They found that the composite membrane delivered a current density of 540 mA/cm^2^ at 0.4 V, while the pure Nafion delivered only 95 mA/cm^2^. One should notice however that the proton conductivity of the composite membrane at 110 °C and 70% RH was as low as 27 mS/cm.

Layer-structured zirconium hydrogen phosphate (Zr(HPO_4_)_2_·H_2_O) (“ZrP”) has a protonic conductivity of the order of 10^−7^–10^−3^ S/cm depending on the phase composition, structure and hydration state [71,72]. A remarkable proton conductivity of 218 mS/cm at 80 °C with 100% RH has been reported for Nafion/ZrP composite as a consequence of enhanced water uptake, which could be explained by the hydrophilicity of ZrP particles, providing additional proton-conducting moieties in the membrane [73]. Yang et al. [72] further suggested that ZrP forms an internal rigid scaffold within the membrane that permits increased water uptake.

The permeation of hydrogen can be substantially reduced by adding layered silicates such as montmorillonite (MMT) or laponite (Lp). Although the proton conductivities of the nanocomposite membranes were lower than that of pure PBI membranes, the performance of the MEAs was higher than the commercial Nafion [74,75].

Heteropolyacids (HPAs) including phosphotungstic acid (PWA) and silicotungstic acid (SiWA) (H_4_SiW_12_O_40_·*n*H_2_O) etc. are known to have high intrinsic proton conductivity as the humidity increases, thus HPAs as inorganic additives have been extensively studied for intermediate-temperature and low-humidity PEMFC applications. However, the proton conductivity of these composite membranes is in general low reportedly because not all protons are available for protonic conduction at higher temperatures even though they move more rapidly [35]. In fact, this protonic conductivity is as stated above probably attributable mainly to adsorbed surface water.

A comparison of the proton conductivities obtained from Nafion-based composite membranes and their PEMFC performance are summarized in Table 2.

## 3. PBI-Based Composite Membranes

Polybenzimidazole (PBI) provides exceptional chemical resistance, high thermo-oxidative stability, and good thermal and mechanical properties at temperatures above 80 °C [84]. The earlier-reported inherent protonic conductivities of PBI are low and contradicting; values such as 10^−12^ S/cm [85], 10^−7^ S/cm [86] and 2 × 10^−4^–8 × 10^-4^ S/cm [87] at RH from 0 to 100% were published, all suggesting that PBI is an insulator without potential use as solid electrolyte in fuel cell applications. Nevertheless, due to the basic character of PBI (p*K*_a_ = 5.23 for 2-phenyl benzimidazole in the salt form [88]), the N–H sites of the imidazole ring associated with PBI can readily combine with strong acids to form a single-phase polymer electrolyte for use as proton conductors [89,90,91]. The proton transport is essentially through proton hopping between two molecules via rearrangement of the hydrogen bonds [92]. Water produced from the cathode can further promote the dissociation of the acid and facilitate proton transport [93]. The type of acids, acid doping level and immersion time, RH and temperature were found to be of special importance for the proton conductivity of the acid-doped PBI membranes. Sulfuric acid (H_2_SO_4_)-doped PBI has been shown to generate the highest conductivity [85,94], however, it has a significant vapour pressure. Besides, the high performance also requires RH of above 50% under H_2_SO_4_ doping level of 9.65 to achieve conductivities of 0.2 S/cm at 150 °C [95]. In the literature, the acid doping level (ADL) is often expressed as the number of H_3_PO_4_ mols per PBI unit. In comparison, it was much easier to dope PBI with phosphoric acid (H_3_PO_4_), forming a dynamic hydrogen bond network so that protons can transport through [96]. A breakthrough was reported by Wainright et al., where they measured a proton conductivity of 22 mS/cm from H_3_PO_4_ doped PBI membrane with a doping level of 5.01 at 190 °C [90].

Since then, H_3_PO_4_ doped PBI type membranes have been studied extensively and seem so far the most successful membranes for application in fuel cells at temperatures up to 200 °C under ambient pressure. For this reason, we will in the present review limit ourselves to H_3_PO_4_-doped PBI composite membranes only. Thermoplastic polymers other than PBI, e.g., poly(vinylalcohol) (PVA), poly(ethyleneimine) (PEI), polyimide (PI), poly(ethyleneoxide) (PEO), poly(ethersulfone) (PES) doped with other types of acids are beyond the scope of this review [19,97,98,99].

### 3.1. H_3_PO_4_ Doped PBI Membrane

**Figure 11:**
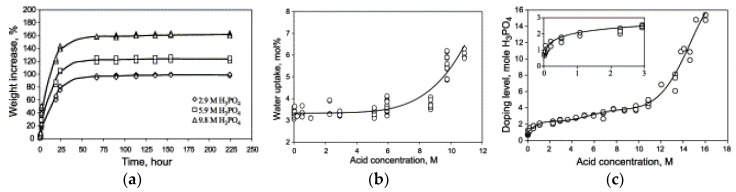
Weight increase as a function of time (**a**); water uptake (**b**); acid doping level of acid-doped PBI membranes at room temperature (**c**). Reprinted with permission from Ref. [100]. Copyright 2004 Elsevier.

As mentioned before, the intrinsic proton conductivity of PBI is negligible and it requires acid doping to facilitate proton transport. After doping with H_3_PO_4_, the associated anions are linked to the PBI polymer, which acts as both a donor and acceptor in proton transfers, allowing protons to move along the anionic chain. As seen from Figure 11a, an immersing time of 50 h is necessary before the weight and volume of the membrane reaches a stable level at ambient temperature. This is due to both water uptake and acid doping. In order to differentiate the contribution of each, the membrane was dried at 110 °C in vacuum for about 5 h until a constant weight was reached. It was assumed that all absorbed water was removed in this way and the gain in volume was due to acid doping alone. Moreover, the amount of absorbed water was very much influenced by the acid concentration (Figure 11b). At low acid concentrations (<5 M), no significant difference in water uptake was observed because the active sites of the imidazole ring are preferably occupied by the doping acid molecules. However, the amount of absorbed water increased significantly at higher acid concentrations (>8 M). In this case, the water uptake is predominated by the excess of hygroscopic acid [93,100]. Li et al. [100] proposed that the H_3_PO_4_ can be classified into “bonded acid” and “free acid”, depending on the acid doping level. Moreover, the water uptake is accompanied by a volume swelling of 20–25 vol%. Figure 11c further shows that when the total acid concentration increases from 2 to 11 M, the “bonded acid” remains more or less constant. In this case, the proton migration takes place via the H^+^ hopping between the N-H site and the phosphate anion. By increasing the acid amount, the amount of “free acid” increases, which is responsible for the increase in protonic conductivity [93]. The protonic conductivity increased from 25 mS/cm to 68 mS/cm at 200 °C and RH of 5% as the acid doping level increases from 2 to 5.6 due to the presence of “free acid”. In brief, the acid doping level related to free acid is the most important parameter that determines the membrane proton conductivity.

In general, a high acid doping level results in high proton conductivity, but excessive acid sometimes leads to the formation of a soft paste, which cannot be processed into a membrane [101,102]. Another disadvantage is acid leaching from the membrane after certain operation time, which decreases the mechanical strength, and reduces the lifetime of PEMFCs severely. Therefore, a balance between acid doping level and membrane stability is required.

Brooks et al. [103] claimed that the absorbed water greatly affects the mechanical properties of PBI. PBI loses its compressive strength at increased water content, which could be due to weakened van der Waals forces and hydrogen bonding interactions as a consequence of polymer swelling. Iwamoto et al. [104] investigated the relationship between the tensile strength of PBI as a function of water content. They found that 10% water absorption resulted in a 25–30% decrease in the tensile strength.

### 3.2. Metal Oxides

Most filler particles used for Nafion-based membranes have also been added into the PBI polymer matrix, which is expected to improve the mechanical strength of the membrane, and also to increase the acid retention capability of the membrane. Herein, we summarize the recent developments around metal oxide-based PBI composite membranes.

Quartarone et al. [105] investigated the role of SiO_2_ with three different morphologies. An acidic silica (HiSilTM T700), a mesoporous SiO_2_ (SBA-15) and an imidazole-functionalised SiO_2_ (SiO_2_-Im) with filler loading ranging between 0–20 wt.% were studied with respect to the physicochemical properties of the H_3_PO_4_-doped PBI composites. SiO_2_-Im fillers were synthesized by means of a standard basic hydrolysis/condensation process, using tetraethoxysilaine (TEOS) and N-(3-triethoxysilylpropyl)-4,5-dihydroimidazole in molar ratio 2:1 as starting materials [106]. The composite membrane increased the acid retention capabilities and facilitated the proton transport across the polymer matrix. In terms of conductivity, the as-prepared PBI/SiO_2_-Im composite membrane was almost independent on the filler concentration, and close to the free acid conductivity [93]. Acid leaching test was performed by washing the as-doped membrane in hot water (80 °C) to remove the free acid. An increase in conductivity is obtained even at relatively small amount of SiO_2_-Im (2 wt.%) at 120 °C and 50% RH [106]. In comparison, an initial conductivity increase was observed for the PBI/HiSilTM SiO_2_ up to 8 wt.%, which is attributed to the acid-base interactions and/or the formation of space charge layers. This is followed by a decrease that could be due to a dilution effect and/or plasticizing effect promoted by an excess of H_3_PO_4_ adsorbed by the fillers [107,108]. Furthermore, the influence of SiO_2_-Im fillers on the MEA performance was studied by Kurdakova et al. [109]. The incorporation of 20 wt.% SiO_2_-Im gave a maximum power density of 83 mW/cm^2^ at 300 mA/cm^2^, which is 20% higher than that of the MEA using pristine PBI polymer. In addition, a reduced charge transfer resistance of the cathode side was observed from electrochemical impedance spectroscopy (EIS).

Devrim et al. [110] prepared H_3_PO_4_-doped PBI with 5 wt.% SiO_2_ by a solid-casting method and reached a high proton conductivity of 102.7 mS/cm at 180 °C and 0% RH. They further fabricated gas diffusion electrodes by an ultrasonic coating technique with 1 mg Pt/cm^2^ catalyst loading for both the anode and cathode in order to test the performance of the membrane in a single HT-PEMFC of a 5 cm^2^ active area at the temperature range of 140–180 °C. A current density of 240 mA/cm^2^ was observed at 165 °C and a cell voltage of 0.6 V. This gave a maximum power density of 250 mW/cm^2^, whereas the pristine-doped PBI measured at the same condition yielded only 185 mW/cm^2^. The application of this composite membrane was not only restricted to PEMFCs and DMFCs, but also for gas separation applications [111].

**Figure 12:**
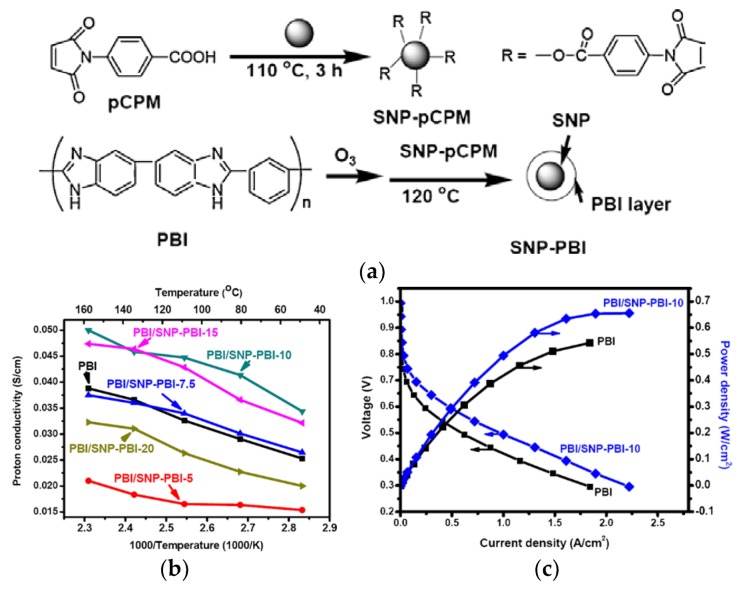
Ozone-mediated process for the synthesis of PBI/SNP–PBI nanocomposite membranes (**a**); proton conductivity of PBI/SNP–PBI membranes with different filler content (**b**); PEMFC polarization curve of PBI compared to PBI/SNP–PBI with 10 wt.% SiO_2_ membranes at 150 °C using dry H_2_/O_2_ as reactant at a flow rate of 0.3 L/min (**c**). Reprinted with permission from Ref. [112]. Copyright 2012 Elsevier.

Suryani et al. [112] synthesized PBI-functionalized SiO_2_ nanoparticles (SNP-PBI) by a previously reported ozone-mediated process [113] using N-(p-carboxyphenyl)maleimide functionalized SNPs (SNP-Pcpm) as precursors to make the PBI chemical bond to the SNP surface (Figure 12a). The synthesized nanofillers were then used for the preparation of the PBI/SNP–PBI nanocomposite membranes for PEMFC application. Interaction between the maleimide groups of SNP-Pcpm and PBI matrix lead to a cross-linked structure of the nanocomposite membrane, which slightly enhanced the membrane’s thermal properties. The addition of the fillers reinforces the membrane due to the enhanced interfacial compatibility between the SNP-PBI and PBI matrix, notified by the increases in their Young’s modulus and the tensile strength. Moreover, it is observed that the acid uptake decreased from 420% to 360% as the filler content increases from 0–20 wt.%. They suggested that the crosslinked structure might depress the swelling effect of the composite membranes as well as acid uptake. This is however in contrast with what has been observed from nanocomposite membranes containing imidazole-functionalised SiO_2_ [106]. Moreover, the membrane with 10 wt.% SNP-PBI fillers showed the highest proton conductivity at 160 °C compared to that of the pristine membrane (Figure 12b). It further showed a maximum power density of 650 mW/cm^2^ in a single H_2_/O_2_ fuel cell test, higher than the 530 mW/cm^2^ obtained from the corresponding pristine PBI membrane (Figure 12c).

PBI–TiO_2_ nanocomposite membranes have been prepared by mixing TiO_2_ nanoparticles with PBI solutions in *N,N*-dimethylacetamide (DMAc) solvent [114,115]. Two wt.% TiO_2_ in PBI reached the highest doping level at 15 mol H_3_PO_4_ per PBI repeat unit and water uptake compared with those obtained from other PBI-based membranes. It further showed high proton conductivity above 100 mS/cm between 125–175 °C and a promising power density of 800 mW/cm^2^ at 150 °C. The influence of TiO_2_ loading (2–16 wt.%) in the PBI membrane was studied by Pinar et al. [116]. They found that membranes containing 2–4 wt.% TiO_2_ exhibited the best properties in terms of acid and water absorption capability and proton conductivity at all operation temperatures. Even larger amounts of fillers did not contribute to any further improvement. The long-term stability of the 2 wt.% TiO_2_ composite PBI membrane was performed in a 150 cm^2^ HT-PEM stack cell, which exhibited an irreversible voltage loss of less than 2% after 1100 h continuously operation [117]. Moreover, the acid leaching from the stack reduced from 2% to 0.6% compared to the standard PBI membrane, indicating that the introduction of fillers enhanced both the durability and stability of the membrane.

**Figure 13:**
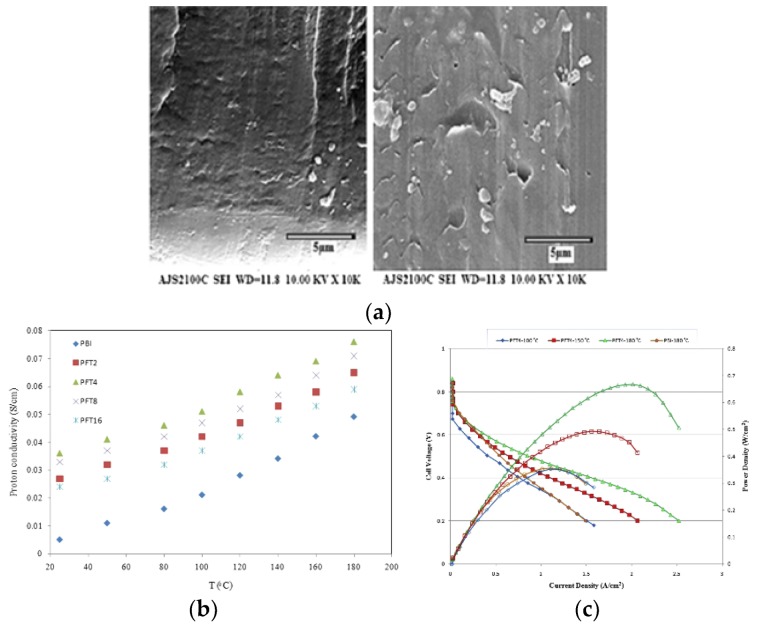
SEM cross-section image of H_3_PO_4_-doped PBI nanocomposite membrane with 4 wt.% and 16 wt.% Fe_2_TiO_5_ nanoparticles (**a**); proton conductivity at RH = 0% (**b**); polarization curves for the single PEMFC of H_3_PO_4_-doped PBI with 4 wt.% Fe_2_TiO_5_ nanocomposite membrane (**c**). Reprinted with permission from Ref. [108]. Copyright 2016 Elsevier.

Moradi et al. [108] synthesized Fe_2_TiO_5_ nanoparticles through a sol-gel process [118], and prepared H_3_PO_4_-doped PBI–Fe_2_TiO_5_ nanocomposite membranes by a solution-casting method for use in HT–PEMFCs. The cross-section SEM image of the nanocomposite membrane containing 4 wt.% and 16 wt.% of Fe_2_TiO_5_ (Figure 13a) showed that in the case of 4 wt.% Fe_2_TiO_5_, the nanoparticles were uniformly dispersed inside the PBI matrix, which is further confirmed by EDX. In the case of 16 wt.% Fe_2_TiO_5_, a significant agglomeration of the nanoparticles was observed. The proton conductivity of the membranes was enhanced by increasing the weight percentage up to 4 wt.% due to the increase of free acid adsorbed by the nanoparticles. Even higher Fe_2_TiO_5_ concentration led to a decrease in proton conductivity due to the agglomeration of nanoparticles as confirmed by SEM. The temperature effect on proton conductivity of the nanocomposites in dry conditions is shown in Figure 13b. A maximum proton conductivity of 78 mS/cm was attained with PBI membranes with a H_3_PO_4_ doping level of 12 and 4 wt.% of Fe_2_TiO_5_ at 180 °C under dry conditions. The good proton conductivity may be explained by the fact that Fe^3+^ cations are located near Ti^4+^ cations in the nanoparticles and increase the acidic properties of these ions. Consequently, the interaction between H_3_PO_4_ and Fe_2_TiO_5_ inside the structure is much stronger, resulting in higher proton conductivity. The 4 wt.% Fe_2_TiO_5_ PBI membrane was also used to prepare a MEA. The single PEMFC performance of the MEA at various temperatures is shown in Figure 13c. As it can be seen, the fuel cell performance increased considerably from 100 to 180 °C, which is due to the faster reaction kinetics and enhanced proton conductivity of the membrane. The highest values of power and current density at 180 °C were 430 mW/cm^2^ and 850 mA/cm^2^, respectively.

Recently, Ozdemir et al. [119] prepared composites of 5 wt.% TiO_2_, SiO_2_ and acidic zirconium phosphate (ZrP, see also next paragraph) nanoparticles in PBI polymer, followed by different H_3_PO_4_ doping levels. The membranes were characterized in terms of their thermal, morphological and mechanical properties. All membranes are thermally stable for temperatures between 130 and 550 °C, their conductivities little affected by the introduction of fillers, and they are reported suitable for use in HT–PEMFCs. The SEM images revealed that SiO_2_ particles were uniformly dispersed in the polymer matrix without sedimentation, contrary to the case for PBI/TiO_2_ composites. The observation is in agreement with Pinar et al., who found that agglomeration occurs for TiO_2_ concentrations higher than 2 wt.% [116]. The effect of fillers on membrane proton conductivities was investigated by EIS. The PBI/SiO_2_ and PBI/ZrP composites showed enhanced properties in terms of acid retention capability and proton conductivity owing to the good interaction between PBI and fillers. High conductivities of 113 mS/cm and 200 mS/cm were achieved for PBI/SiO_2_ and PBI/ZrP, respectively, at 180 °C and non-humidified conditions. These values are much higher than the previously reported ones [106], and the corresponding activation energies were only 23.8 kJ/mol and 19.4 kJ/mol. As mentioned above, the conductivity of PBI membranes increases with acid doping level on one hand, but sacrifices the mechanical strength on the other hand. The tensile strength of the pristine PBI membrane was highest (127 MPa) among all as anticipated, while PBI/TiO_2_ showed the lowest tensile strength (85.6 MPa) due to the uneven distribution of TiO_2_ particles. The specific interactions between SiO_2_ and PBI generate a mechanism for reinforcement, consequently, the elongation at break increased significantly.

### 3.3. Solid Acids—Heteropolyacids (HPA), Zirconium Phosphate (ZrP), and Caesium Salts of HPA

Hydrated heteropolyacids (HPAs) with the general formula of H_x_AM_12_O_40_·H_2_O, where A stands for P or Si, and M indicates W or Mo, are called solid acids and display high conductivity and acidity. The proton conductivity, mainly by the Grotthuss mechanism [120], is mainly confined to adsorb surface water layers.

The use of PBI membranes with two different HPAs, namely phosphotungstic acid, H_3_PW_12_O_40_·*nH*_2_O (PWA) and silicotungstic acid, H_4_SiW_12_O_40_·*nH*_2_O (SiWA) for fuel cell application was first reported by Staiti et al. [121,122]. A maximum conductivity of 3 mS/cm was obtained under fully humid conditions at 100 °C with 60 wt.% PWA/SiO_2_ in PBI, and the proton conductivity remained more or less constant as the temperature increased to 150°C. This conductivity is however too low for fuel cell applications. Inspired by this, Aili et al. developed a novel composite membrane based on an PWA-impregnated mesoporous SiO_2_ functionalized H_3_PO_4_-doped PBI membrane, PBI/PWA-meso-SiO_2_ [123]. The stability of the acid-doped PBI membrane-based fuel cell enhanced substantially after the addition of PWA-meso-SiO_2_ fillers, most possibly due to the formation of the phosphosilicate phase between H_3_PO_4_ and mesoporous SiO_2_.

He et al. [93] cast PBI composite membranes by dissolving commercial PWA and SiWA in DMAc, followed by mixing 5 wt.% PBI in DMAc, and immersion in H_3_PO_4_ of different concentrations in order to obtain sufficient proton conductivity. However, the conductivity after introducing 30 wt.% of PWA and SiWA in PBI was slightly lower than that of the H_3_PO_4_-PBI membrane under the same conditions. A possible explanation is that the HPAs diluted the density of the acid groups that provide transport for protons. Verma et al. emphasized in their paper that SiWA neutralized with NaOH prior to acid doping of PBI avoids agglomeration and leads to well-defined proton pathways, which improved the proton conductivity [124]. The same group prepared zirconium hydrogen phosphate Zr(HPO_4_)_2_·*n*H_2_O (ZrP) by mixing zirconyl chloride (ZrOCl_2_) with orthophosphoric acid with a mole ratio of 1:2, following the procedure described by Staiti et al. [121]. A similar procedure was also employed by Qian et al. [125]. In both cases, a washing step was necessary to eliminate the remaining Cl^−^ and the excess acid inside the crystal. The composite membrane with 15 wt.% ZrP was found to exhibit slightly higher conductivity than that of the 20 wt.% ZrP at all temperatures, but the reasons for this behaviour are not clear. A conductivity of 96 mS/cm with corresponding activation energy of 11.9 kJ/mol was obtained at 200 °C and 5% RH, a conductivity value that is comparable to that of Nafion membrane at 80 °C and 100% RH. For composite PBI/ZrP membranes, different ionic species such as H_3_O^+^, H_2_O, PO_4_^3−^, and P–O and P–OH species can exist by bonding with ZrP inside the structure or at the surface, which can aid the proton conductivity compared with that of PBI membranes [93,126].

Yamazaki et al. [127] prepared a zirconium tricarboybutylphosphonate Zr(PBTC) powder by using 2-phosphonobutane-1,2,4-tricarboxylic acid in place of orthophosphoric acid, and dispersed the powder in a PBI solution of DMAc. The presence of Zr(PBTC) grains were reported to inter-connect with each other, providing conduction paths via the PBI polymer. The proton conductivity of the composite membrane with 50 wt.% Zr(PBTC) increased with a factor of 2 after H_3_PO_4_ treatment and post-sulfonation thermal treatment, suggesting the possible use of the Zr(PBTC)/PBI as an electrolyte material for fuel cells.

Li et al. [128] prepared Cs_2.5_H_0.5_PMo_12_O_40_ (CsPOMo) by mixing phosphomolybdic acid with caesium carbonate (Cs_2_CO_3_), and blended it with PBI to form a PBI/CsPOMo composite membrane. A chemical bond between CsPOMo and PBI was formed, as indicated by ^31^P NMR analysis. Good thermal stability and high proton conductivity of >150 mS/cm were achieved after doping the composite with H_3_PO_4_. It is noteworthy that the conductivity of the PBI/CsPOMo membrane without H_3_PO_4_ was only 0.04 mS/cm at 150 °C and 0% RH, implying an important role of H_3_PO_4_ and water.

**Figure 14:**
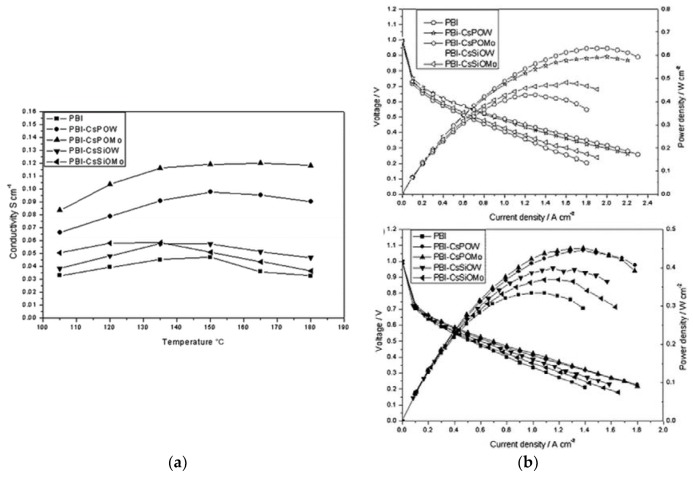
Proton conductivities of PBI composite membrane containing 30 wt.% CsHPA at ADL = 4.5, RH < 1% (**a**); polarization and power density curves of a PEMFC operated at 150 °C with H_2_/O_2_ atmospheric pressure (upper plot) and H_2_/air atmospheric pressure (lower plot) (**b**). Reprinted with permission from Ref. [129]. Copyright 2011 Royal Society of Chemistry.

A similar study was carried out by Xu et al. [129], where they synthesized four Cs salts of heteropolyacids, denoted as Cs_X_H_3–X_PMo_12_O_40_ (CsPOMo), Cs_X_H_3–X_PW_12_O_40_ (CsPOW), Cs_X_H_4–X_SiMo_12_O_40_ (CsSiOMo) and Cs_X_H_4–X_SiW_12_O_40_ (CsSiOW) to form composite membranes with PBI. All four Cs salts were nanoparticles of around 100 nm. Subsequently, acid loading was conducted by immersing the composite membrane in H_3_PO_4_ solution of different concentrations for a certain time. All the composite membranes showed conductivity values (e.g., 47 mS/cm at 150 °C and 0% RH) to be higher than H_3_PO_4_/PBI membranes. Membranes with CsPOMo and CsPOW achieved significantly higher conductivity than CsSiOMo and CsSiOW. On the contrary, lower mechanical strength was observed from CsHPA with P atoms. The reason for the difference in conductivities of the P and Si-based CsHPA when formed as acid-doped PBI composite is still unknown. One possible explanation is the smaller particle size with P atoms (observed from SEM images), which will provide a more active surface area and potentially adsorb more acid. Increasing the CsHPA content in the composites also led to an enhancement of the proton conductivity. Figure 14a shows the conductivity of the 30 wt.% CsHPA/PBI membranes at ADL of 4.5 as a function of temperature under anhydrous conditions. The highest conductivity of 120 mS/cm was achieved from CsPOMo at 150 °C, which is in good agreement with Li et al. [128]. The results imply that this membrane is a promising material for high-temperature fuel cells. The test of the composite membranes in an actual fuel cell is shown in Figure 14b. All membranes have low gas permeability as the open circuit voltages were all above 0.95 V. The enhancement of the cell performance from the composite membranes was attributed to the higher proton conductivity and stronger acid retention properties. CsPOMo gave a maximum power density of around 600 mW/cm^2^ with H_2_/O_2_ gas feeds at atmospheric pressure.

### 3.4. Carbon-Based Materials

**Figure 15:**
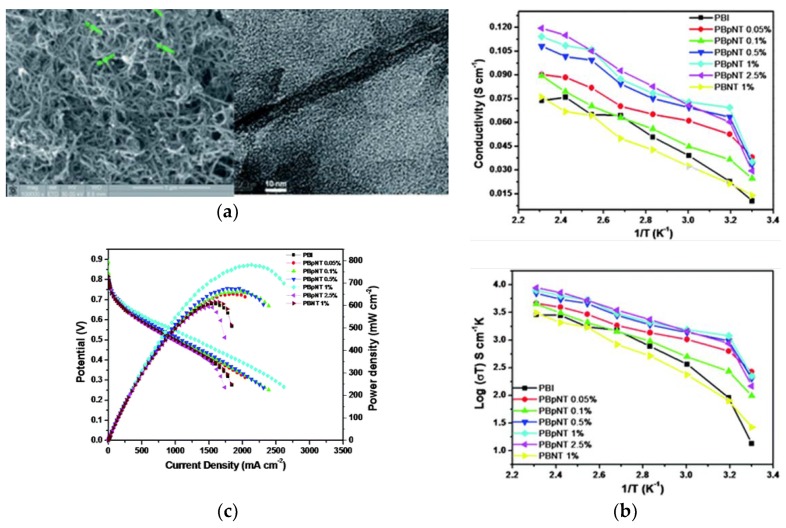
SEM image of p-MWCNTs and TEM image showing the distribution of p-MWCNTs inside the PBI matrix (**a**); proton conductivity and Arrhenius plots of the different composite membranes and pristine PBI (**b**); fuel cell polarization curves measured by supplying dry H_2_/O_2_ with a flow rate of 0.2 standard liter per minute (slpm) at 140 °C (**c**). Reprinted with permission from Ref. [132]. Copyright 2011 Royal Society of Chemistry.

Both single-walled nanotubes (SWCNTs) and (MWCNTs) have been demonstrated to have superior mechanical, thermal and electronic properties, which make them attractive as advanced filler particles in composites [51]. The addition of CNTs to the polymer has shown improved tensile strength, Young’s modules, and elongation at break, which was recently suggested to be attributed to the restriction of the polymer chain movements due to the effectual interfacial interactions such as H-bonding and π-π interactions [130]. Nevertheless, precautions must be taken when using CNTs in PEM, as the electronic conductivity may short circuit PEMFCs. Kannan et al. [131] demonstrated a dual functionalization strategy to incorporate phosphonic acid on the sidewalls of MWCNTs in order to make a composite with H_3_PO_4_-doped PBI membranes. The SEM image in Figure 15A (left) shows that the diameter of p-MWCNT is between 14–20 nm. The authors emphasized that the microwave treatment was critical for improved solubility hence the homogeneity of the membrane. Individual MWCNTs are observed instead of bundles, as seen from the TEM images. Composite PBI membranes with low levels of p-MWCNT (0.05–2.5 wt.%) have been prepared to avoid possible short circuit and agglomeration [132]. The composites were subsequently immersed in H_3_PO_4_. The TGA of the resulting membranes led to similar patterns under N_2_ atmosphere between 50 and 700 °C, indicating the addition of CNTs did not cause any significant thermal degradation. The proton conductivity and the corresponding Arrhenius plots of the prepared membranes (PBpNT) were compared to a composite PBI with un-functionalized MWCNT (PBNT 1%), as shown in Figure 15B. The conductivity increased with increasing amounts of p-MWCNTs in the composite, except for the PBpNT 0.05 wt.%. The best composite membrane achieved almost half an order of improvement in the proton conductivity upon doping with H_3_PO_4_ (110 mS/cm for PBpNT 1%*)*. The conductivity value was in good agreement with the value reported by Suryani et al. [133], where they prepared MWCNT through an ozone-mediated process and used it as a filler to make PBI/MWCNT composite membranes. The poor performance of PBNT 1% was due to the absence of phosphonate groups [132]. The drop in the activation energy further suggests the synergistic role of the p-MWCNTs in facilitating proton conduction. With respect to the fuel cell performance (Figure 15C), a maximum power density of 780 mW/cm^2^ was attained from composite PBI membrane with 1 wt.% p-MWCNT in comparison with 600 mW/cm^2^ for the pristine PBI membrane under identical conditions.

The incorporation of small amounts of graphene oxide (GO) in the PBI membrane showed positive effects in terms of acid retention, proton conductivity, tensile strength and toughness. High-performance H_3_PO_4_-doped PBI/GO composite membranes have been prepared by Üregen et al., showing a high conductivity of 129.7 mS/cm at 165 °C at a GO content of 2 wt.% [134]. This is because hydrogen bonds in GO form acidic functional groups like carboxylic acid and epoxy oxygen, which provide pathways for proton hopping [135]. The GO in the PBI membrane is suggested to form well-connected channels that facilitate proton hopping through the membrane, thus improving the conductivity. Nonetheless, the formation of acidic functional groups in GO degrades the membrane and leads to reduced proton conductivity at temperatures above 165 °C. The maximum power density reached from a single PEMFC with a 5 cm^2^ active area constructed with PBI/2 wt.% GO was 378 mW/cm^2^ operated at ambient pressure and 165 °C. An open circuit voltage of 0.95 V implies that the membrane possessed low hydrogen crossover. At a constant current of 200 mA/cm^2^, the composite membrane loss was about 3.8% after 500 h operation, while the H_3_PO_4_-doped PBI membrane showed 8.3% loss. This suggests that GO can trap more acid molecules and prevent acid leaching out of the membrane.

**Figure 16:**
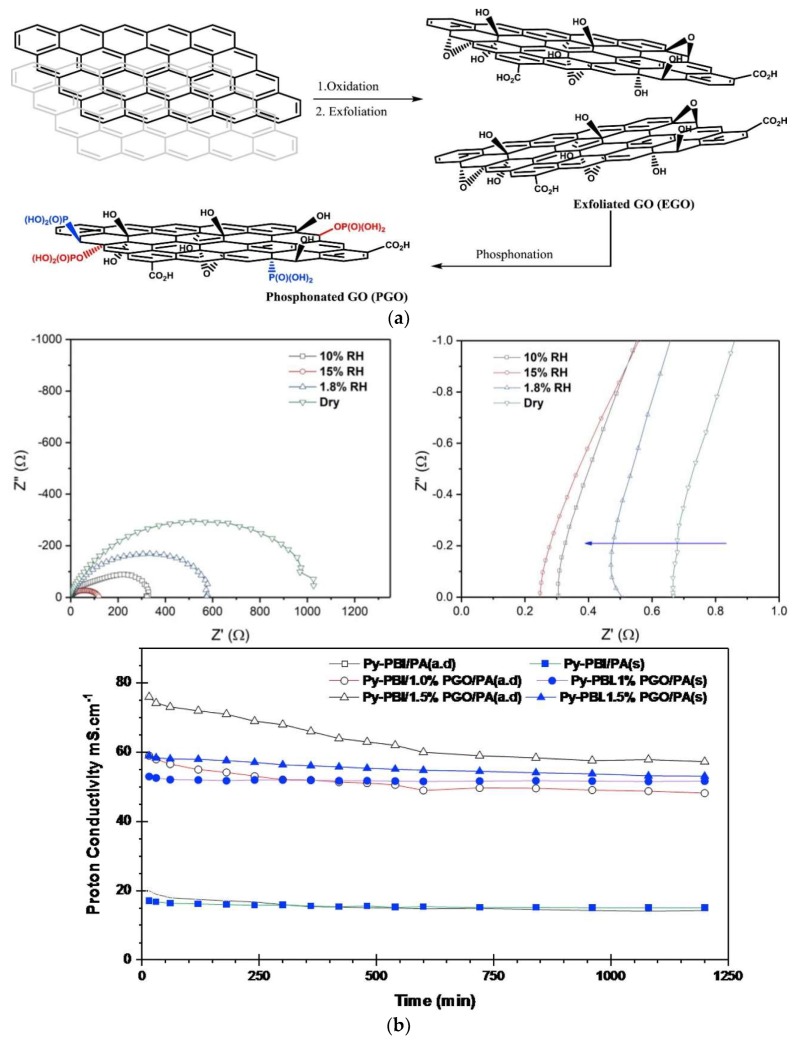
Preparation of functionalized graphene oxide (**a**); Nyquist plot of H_3_PO_4_-doped py-PBI/1.5 wt.% pGO under various RH at 120 °C; zoom-in in the high frequency intercept; and proton conductivity of the py-PBI composite membranes with different filler content as a function of testing time at 140 °C and 0% RH (**b**). Reprinted with permission from Ref. [136]. Copyright 2019 Elsevier.

Phosphonated graphene oxide (PGO) embedded in 2,6-pyridine functionalized PBI (py-PBI) at different H_3_PO_4_ doping levels has been evaluated as HT-PEM material [136]. The pGO was prepared from graphite in a three-step procedure involving oxidation, exfoliation and phosphonation (Figure 16A), followed by a solution casting method to fabricate the composite membrane. After doping with H_3_PO_4_, the best proton conductivity was obtained from the py-PBI/1.5 wt.% PGO membrane, showing a conductivity value of 76.4 mS/cm at 140 °C under anhydrous condition, whereas the py-PBI membrane without filler showed a value of 19.6 mS/cm under similar conditions. The improvement could be explained by the inherent strong hydrogen bonding between localized phosphonic acid groups of GO and imide groups of pyPBI that help to form a network for efficient proton transport. The proton conductivity measurement was further conducted for 20 h in order to investigate the durability of the composite membranes. As can be seen in Figure 16B, a significant drop in proton conductivity was evidenced from the one with 1.5 wt.% PGO during the first 5 h of operation, then remained more or less constant throughout the rest of the experiment. In comparison, the membrane with 1 wt.% PGO seems to be more stable. The highest performance in a fuel cell was also achieved from the same membrane, with a peak power density of >359 mW/cm^2^ at 120 °C and 0% RH, which is 75% higher than the membrane without PGO.

Xue et al. [137] prepared graphite oxide/PBI from 3,3′-diaminobenzidine and 5-tert-butyl isophthalic aicd (GO/BuIPBI) and isocyanate modified GO/BuIPBI (iGO/BuIPBI) composite membranes, followed by H_3_PO_4_ loading for HT-PEMFCs. At 140 °C with no humidity, the proton conductivities of the H_3_PO_4_-doped BuIPBI, 5 wt.% iGO/BuIPBI and 5 wt.% GO/BuIPBI were 12, 16 and 27 mS/cm, respectively. Interestingly, the 10 wt.% iGO/BuIPBI demonstrated better proton conductivity than the 15 wt.% iGO/BuIPBI as an excessive amount of iGO might agglomerate and block the proton conduction paths.

OPBI/GO composite with only 0.3 wt.% GO was fabricated by a solvent-exchange method [138]. It showed a 17% increase in Young’s modulus, 33% increase in tensile strength and 88% enhancement in toughness compared to pure OPBI membranes, which may find application in fuel cells.

### 3.5. Metal Organic Frameworks (MOFs)

**Figure 17:**
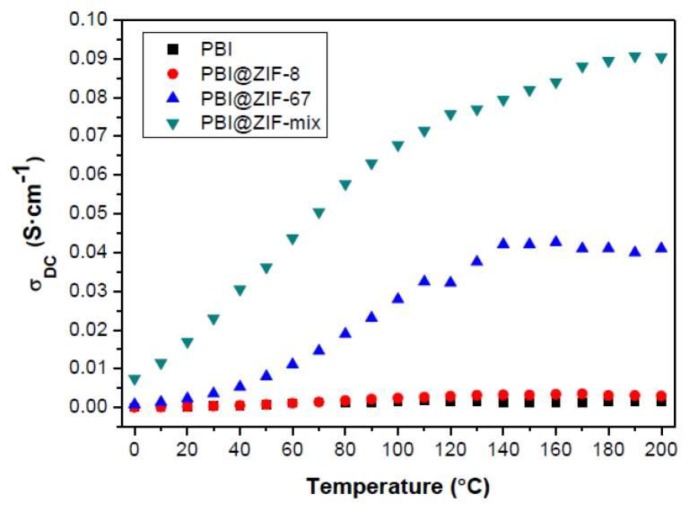
The temperature-dependent proton conductivity of H_3_PO_4_-doped PBI membrane and PBI composite membranes with 5 wt.% of ZIFs. Reprinted with permission from Ref. [57] under a Creative Commons Attribution (CC-BY) License. Copyright 2018 MDPI.

In the past few years, the use of metal organic frameworks (MOFs) as fillers in PEMs has received massive attention. Zeolitic imidazolate frameworks (ZIFs) is a unique subgroup of MOFs that demonstrated high porosity in addition to being chemically and thermally stable [139]. Recently, Escorihuela et al. prepared a Zn-based ZIF-8, a Co-based ZIF-67, and a binary mixture of the two (represented as ZIF-mix), which have been embedded in the PBI matrix for the preparation of PBI composite membranes containing 5 wt.% of these fillers [57]. The as-prepared composites were characterized after immersing in H_3_PO_4_. The phosphonate anion seemed to attract Co more than Zn, as the acid uptake from PBI@ZIF-67 is higher (166%) after the same immersion time. Proton conductivity measurements of the membranes were performed at 0–200 °C under anhydrous conditions (Figure 17). Significantly enhanced proton conductivity was observed for the ZIF-67 and ZIF-mix with increasing temperature. A plateau around 140 °C was evidenced for the composite with ZIF-67, which may be due to the evaporation of H_3_PO_4_. The proton conductivity for acid-doped PBI@ZIF-67 reached 41 mS/cm at 200 °C, but an even higher proton conductivity of 91 mS/cm was achieved from the hybrid PBI@ZIF-mix membrane. This increase implies a synergistic effect between the two MOFs, resulting in more consecutive hydration channels, as observed with other PEM composites with fillers such as UiO–66–SO_3_H/UiO–66–NH_2_ [80]. The proton conductivities obtained in this work were among the highest reported for MOF-containing HT–PEMs measured under anhydrous conditions [140]. They further proposed that the proton conductivity in ZIF-containing PBI composites was mainly based on a Grotthuss mechanism, while the proton transfer from the surface of ZIF to the next ZIF by H_3_PO_4_ as proton carrier can be explained by the vehicle mechanism. A test of fuel cell performance based on ZIF-containing PBI composite membranes was however not conducted in their work.

### 3.6. Clays

**Figure 18:**
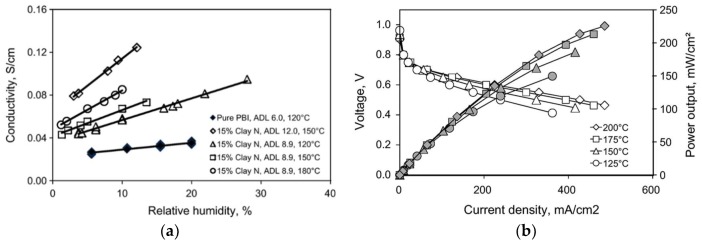
Conductivity of PBI membrane and PBI/clay N composites as a function of relative humidity at different temperatures (**a**); polarization curve for a fuel cell with PBI/5 wt.% clay L composite membranes (ADL = 8.2) obtained at different temperatures (**b**). Reprinted with permission from Ref. [141]. Copyright 2011 Elsevier.

Two types of organically-modified nanoclays, imidazole salt-modified laponite RD (referred to as clay L) and dequalinium chloride salt-modified laponite RD (clay N) were synthesized by ion-exchange reactions with ammonium and pyridinium salts, and used for the preparation of PBI composite membranes [141]. Figure 18a shows the conductivity as a function of RH at different temperatures. At a clay content of 15 wt.%, it was clear that the conductivity depends on the H_3_PO_4_ doping level, RH and temperature. At ADL = 6, the PBI membrane without fillers exhibited a proton conductivity of about 30 mS/cm at 120 °C and 20% RH, which is in good agreement with previously reported results [93]. The highest proton conductivity was obtained from the PBI composite membrane with 12 wt.% clay N at ADL of 12 (120 mS/cm at 150 °C and 12% RH). The effect of the clay content on the proton conductivity of the PBI membranes was not investigated. They further showed that the hydrogen permeability of the composite membrane was five times lower than that of the unfilled PBI membrane at 150 °C. The fuel cell performance with the PBI-clay composite containing 5 wt.% clay L at different temperatures is presented in Figure 18b. At 175 °C with a catalyst loading of 0.66 mg Pt/cm^2^ for each electrode, a current density of 210 mA/cm^2^ was achieved at a cell voltage of 0.6 V.

Nanocomposite polymers consisting of two modified organoclays, namely, montmorillonite (OMMT) and kaolinite (OKao) in poly(4,4′-diphenylether-5,5′-bibenzimidazole) (OPBI) were prepared by dispersing the silicate layers in the OPBI matix by a solution blending method [142]. The morphology of the composites are dependent on the structure of the clays. In the case of OPBI/OMMT, an exfoliated structure is obtained, whereas intercalated morphology was achieved from OPBI/OKao. Loading of both organoclays enhanced the thermal stability of OPBI compared to the pristine one. The exfoliated structure for OMMT seemed more beneficial in terms of thermal stability due to the higher dispersion of the clay into the OPBI matrix. The nanoparticles in the OPBI polymer shielded the polymer chains from the attack of oxidative radicals (OH* and HOO*) and enhanced the oxidative stability of the membrane. At a high H_3_PO_4_ doping level, the proton conductivity increased with increasing temperature and clay content, and reached 72 mS/cm and 92 mS/cm at 160 °C, respectively, for OPBI/OKao and OPBI/OMMT with 7 wt.% fillers. The authors suggested that the proton conductivity was supported by a continuous “forming–breaking–forming” process of the hydrogen bonds of OPBI and the organoclays with the acid, which promotes the proton transfer in the composites. The very small activation energies (~8 kJ/mol) however suggest a faster (for instance liquid state) proton conduction process. The nanocomposite membranes have not been tested in HT-PEMFCs. Moreover, long-term stability tests are needed to evaluate the feasibility of this composite membrane.

Some characteristic parameters of different H_3_PO_4_–PBI-based composite membranes, in particular those that have been tested in PEMFCs at various conditions, are summarized in Table 3.

## 4. Sulfonated PEEK-Based Composite Membranes

The use of sulfonated hydrocarbons as polymer backbones has also been widely studied in order to form effective water channels, with the most common one being the sulfonated aromatic hydrocarbon polyetheretherketone (SPEEK). SPEEK has certain advantages over Nafion as it is also commercially available (Victrex@PEEK) at a much lower cost. In addition, the SPEEK structure allows the introduction of polar sites that help to increase water uptake [98]. In comparison to Nafion, the water channels in SPEEK are narrower, less separated and more branched with more dead-end channels. This leads to more hydrophilic/hydrophobic interfaces, which result in a larger average separation of neighbouring sulfonic acid functional groups [147].

The properties of SPEEK membranes synthesised directly from the sulfonated monomers are greatly improved in comparison to those of the post-sulfonated PEEK membranes [148]. SPEEK-based PEM with a long-term stability of at least 1000 h (in particular ≥3000 h) at a voltage range from 0.4 V to 1.1 V indicate that SPEEK-based membranes are durable enough under fuel cell operations [149]. Since the proton conductivity of SPEEK depends on several parameters, such as the choice of casting solvent, sulfonation procedure, degree of sulfonation, RH and temperature, large inconsistencies in proton conductivity are evidenced in the literature, especially at low temperatures (~80 °C). A summary of such data can be found in Ref. [150].

SPEEK composites containing 10% amorphous SiO_2_, 30% ZrP or 40% amorphous zirconium phosphate sulfophenylphosphonate have been successfully prepared. All composites exhibited protonic conductivities in the range of 30–90 mS/cm at 100 °C under 100% RH, suggesting their potential as alternative PEM materials to Nafion for PEMFCs up to 120 °C [151].

Novel nanocomposite membranes based on SPEEK and iron titanate, Fe_2_TiO_5_, were prepared by the dispersion of Fe_2_TiO_5_ nanoparticles into SPEEK solution with the solution casting method [152]. Composite membranes with 1 wt.% Fe_2_TiO_5_ showed the highest proton conductivity of 96 mS/cm at 80 °C, which is 65.5% and 6.6% higher than that of pristine SPEEK membrane and Nafion 117 membrane, respectively. This composite membrane also showed a single fuel cell performance of peak power density of 188 mW/cm^2^ at 80 °C under 90% RH.

The use of a natural zeolite in SPEEK, i.e., SPEEK/analcime composite membrane with 5, 10, 15, 25 and 35 wt.% analcime were synthesized for fuel cell applications [153]. Measurements performed under 100% RH showed that the proton conductivity of SPEEK increased with increasing temperature until 80 °C because of faster proton diffusion. However, a further increase in temperature led to a dramatic decrease in conductivity due to the membrane degradation. Such a decrease in proton conductivity was observed for SPEEK/10 wt.% analcime composite membrane at a temperature close to 100 °C. A maximum conductivity of 401.6 mS/cm was reached at 90 °C, with a corresponding activation energy of 15.1 kJ/mol. A similar trend and high conductivity have been reported for Nafion/15 wt.% analcime composite membrane [79]. They concluded that the hydrophilicity and protons inside the connected water channels of the zeolite are expected to be responsible for the superior conductivity. Nevertheless, these conductivity values are considerably higher than most of the previous published values obtained from SPEEK composite membranes, where some of them are summarized in Table 4. It would be interesting to know the performance of such a composite membrane in an actual PEMFC or PEME.

**Figure 19:**
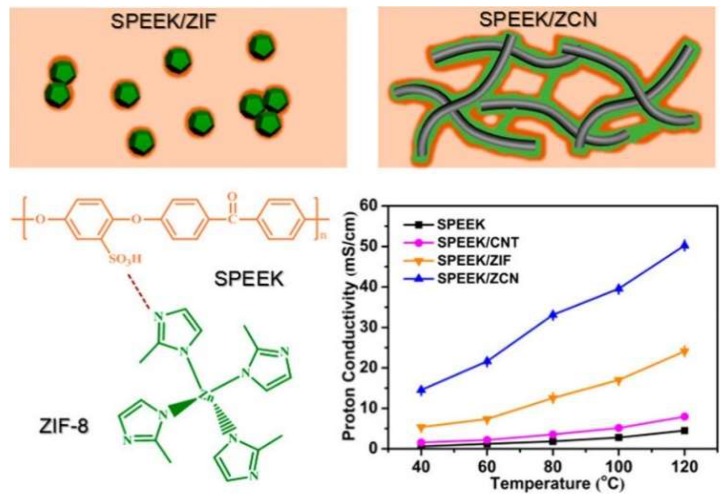
Interaction between SPEEK and ZIF-8, and the schematic illustration for the interface of SPEEK/ZIF and SPEEK/ZCN composite membranes. Reprinted with permission from Ref. [59]. Copyright 2017 American Chemical Society.

Sun et al. [59] synthesized two-dimensional ZIF-8/CNT hybrid cross-linked networks (ZCN) from ZIF-8. The ZCN was then introduced into SPEEK, and three SPEEK composite membranes were prepared, namely SPEEK/ZCN, SPEEK/CNT, and SPEEK/ZIF-8. Significant enhancement in the proton conductivity was observed from the SPEEK/ZCN membrane, which benefited from the 2D network. In particular, as shown in Figure 19, the composite with 2.5 wt.% ZCN achieved a proton conductivity of 50.24 mS/cm at 120 °C at 30% RH, which was 11.2 times better than the recast SPEEK membrane (4.5 mS/cm) and 2.1 times higher than the SPEEK/ZIF membrane (24.1 mS/cm). This study reveals that the proton conductivity of SPEEK-based composite membranes can be enhanced by creating superstructures of MOFs rather than modifying the chemical component, which may be applied to other types of MOFs as well.

S–UiO–66 is another stable MOF with functional groups of –SO_3_H in its ligands that have been grown on graphene oxide (GO) hybrid nanosheets via a facile in-situ procedure, and then used for the fabrication of the SPEEK/S–UiO–66@GO composite membrane [62]. Taking the advantage of the tethering effect of GO surface and interconnection among S–UiO–66 grains, S–UiO–66 was well dispersed onto GO nanosheets, which effectively eliminated the agglomeration of S–UiO–66 in the SPEEK matrix [68]. The resulting composite membrane presented a significant increase in proton conductivity, 268 mS/cm at 70 °C and 95% RH and 16.57 mS/cm at 100 °C and 40% RH, which is 2.6 and 6 times than that of the recast SPEEK membrane under the same condition. The high proton conductivity indicates the great potential of this composite membrane for fuel cell application.

**Figure 20:**
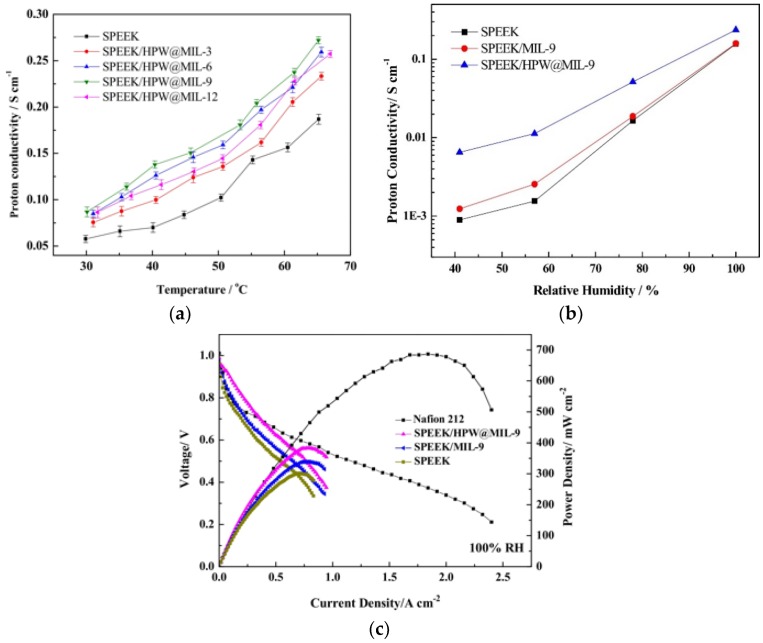
Proton conductivity of SPEEK/HPW@MIL101 membranes with different filler content at 100% RH under different temperatures (**a**); proton conductivity of the membranes with 9 wt.% HPW@MIL101 under different RHs (**b**); single H_2_/O_2_ fuel cell performance (**c**). Reprinted with permissions from Ref. [63]. Copyright 2017 Elsevier.

Zhang et al. presented a novel approach to in-situ synthesize and imbue phosphotungstic acid into the cavity of MIL-101 (Cr) using Na_2_WO_4_·2H_2_O and Na_2_HPO_4_ as precursors (HPW@MIL101), which are then used for the preparation of SPEEK/HPW@MIL101 nanohybrid membranes [63]. In this way, nanochannels are formed both in the cavity of MIL101 and at the interface of HPW@MIL101 and SPEEK, increasing the chance for proton transport. At 9 wt.% HPW@MIL101, the composite membrane exhibited a proton conductivity of 272 mS/cm at 65 °C and 100% RH, which is 45.5% higher than the pristine SPEEK (187 mS/cm) (Figure 20a). When the relative humidity was decreased to 40%, the corresponding proton conductivity for the SPEEK/HPW@MIL101 membrane at 60 °C was 6.51 mS/cm, 7.25 times higher than the pristine (0.898 mS/cm). The pristine SPEEK membrane was very sensitive to changes of RH, while the introduction of phosphotungstic acid retained much of the conductivity, in particular under low relative humidities (Figure 20b). Furthermore, the single H_2_/O_2_ fuel cell performance tested at 60 °C was also improved after the introduction of fillers. As it can be seen in Figure 20c, a power density of 383 mW/cm^2^ at 100% RH was achieved from the composites with 9 wt.% HPW@MIL101, which is 27% higher than that of pristine SPEEK (280 mW/cm^2^).

Finally, composite membranes based on SPEEK/PEEK-BI displayed superiority in terms of oxidative stability due to the presence of benzimidazole groups, but the proton conductivity was lower than that of the corresponding pristine SPEEK at 80 °C under fully hydrated conditions, which was a result of reduced water uptake and swelling ratio [154].

A summary of the SPEEK-based composite membranes is given Table 4.

**Table 4:** Comparison of proton conductivities of sulfonated PEEK-based composite membranes and their PEMFC performance.

Membrane	Water Uptake	Conductivity/Temp/%RH/ Activation Energy	Maximum Power Density in PEMFC	Ref.
SPEEK/9.6 wt.% WC-SiW	6.95 ± 0.08@30 °C	50 mS/cm at 95 °C and 85% RH	Not reported	[155]
		11.2 kJ/mol		
SPEEK/2.5 wt.% ZCN	19.4%@30 °C	50.24 mS/cm at 100 °C and 100% RH	Not reported	[59]
		15.93 kJ/mol		
SPEEK/10 wt.% Analcime	31%@25 °C	401.6 mS/cm at 90 °C and 100% RH	Not reported	[153]
		15.1 kJ/mol		
SPEEK/10 wt.% S-UiO-66@GO	30 wt.%@100 °C	268 mS/cm at 70 °C and 95% RH	Not reported	[62]
		9 kJ/mol		
SPEEK/2.5 wt.% ZrO_2_	20 wt.%@25 °C	40 mS/cm at 90 °C and 100% RH	Not reported	[156]
SPEEK/40 wt% polysilsesquioxane		142 mS/cm at 120 °C and 100% RH	Not reported	[157]
SPEEK/7.5 wt.% sul-MIL101	30%@25 °C	306 mS/cm at 75 °C and 100% RH	Not reported	[58]
SPEEK/1 wt% Fe_2_TiO_5_	61%@25 °C	96 mS/cm at 80 °C and 90% RH	80 °C and RH = 90% 188 mW/cm^2^	[152]
		10.8 kJ/mol		
SPEEK/5 wt.% s-GO	60%@30 °C	55 mS/cm at 80 °C and 30% RH	80 °C and RH = 30% 378 mW/cm^2^	[64]
		22.21 kJ/mol		
SPEEK/SFMC/5 wt.% GO	49.15@90 °C	111.9 mS/cm at 90 °C and 100% RH	70 °C and RH = 100%	[158]
		21.31 kJ/mol	528.01 mW/cm^2^	
SPEEK/9 wt.% HPW@MIL101	29%@25 °C	272 mS/cm at 65 °C and 100% RH	60 °C and RH = 100%	[63]
		6.51 mS/cm at 60 °C and 40% RH	383 mW/cm^2^	

## 5. Mixed Electron-Proton Conducting Composite Membranes for PEMFCs and Beyond

A critical parameter when designing PEMFC electrodes is the formation of a “three-phase” boundary among the substrate-supported catalyst, polymer and reactants. This requires that the gas/liquid, ion conduction and the electrocatalytically active and electronically-conducting phases must be present. If the Nafion content is low, then not all the catalyst particles are connected to the membrane, and therefore the catalyst utilization is reduced [159]. In the case where the Nafion content is too high, then the catalyst particles have poor electrical contact, resulting again in low catalyst utilization and cell efficiencies [160]. Nafion is an insulator so there is an optimal Nafion content that allows good ion conduction, but at the same time the catalyst particles have good electronic conduction too [161,162,163,164].

**Figure 21:**
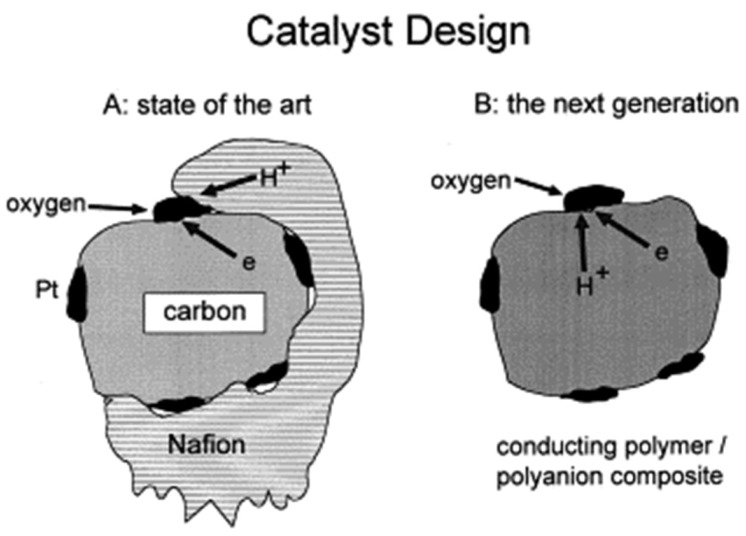
The commonly used electrode configuration (**A**) and the preferred design (**B**) in which the electronic conduction is added in parallel to the protonic one. Reprinted with permission from Ref. [166]. Copyright 1998 Elsevier.

A promising alternative is to mix or replace Nafion in the catalyst ink with an electron-conducting polymer (ECP). In this way, electronic conductivity is introduced in parallel with the protonic one [165]. This is nicely depicted by Qi Z. et al. [166], who replaced Nafion by a mixture of poly(pyrrole)-polystyrenesulphonate (PPY/PSS), see Figure 21. In dry conditions, the electronic conductivity of the PPY/PSS composite membrane was 3 S/cm, which after deposition of the Pt particles was decreased to 0.3 S/cm. This decrease was attributed to the hydrogenation of the PPY during the Pt formation. The protonic conductivity of a 1-μm-thick film in 1 M H_2_SO_4_ at 0.15 V vs. SCE at room temperature and atmospheric pressure was 0.23 S/cm. The stability of the electrode was very good after 70 h of operation at +0.1 V vs. SCE and a current density of around 30 mA/cm^2^. It should be noted that the authors did not assemble a full PEMFC, therefore one could expect a different performance of such an electrode.

Among the most studied ECPs are poly(pyrrole) (PPY), poly(aniline) (PANI) and poly(3,4-ethylenedioxythiophene) (PEDOT) [167]. The synthesis of these ECPs can be conducted by chemical, enzymatic and electrochemical methods. A comprehensive review article regarding the synthesis and applications of ECPs can be found in Ref. [168]. PPY in combination with Nafion has been used in PEMFCs [169,170,171], but the main disadvantages are the degradation PPY by H_2_O_2_, the unfavourable redox potentials and the low permeability of O_2_ gas [165]. It should be noted though that PPY/Nafion composites have been extensively studied in direct methanol fuel cells (DMFCs), where the methanol crossover is heavily impeded by the addition of PPY in the Nafion matrix [172,173,174,175,176,177,178,179]. Although the mechanism behind the reduced methanol permeation is beyond the scope of this review, it is worth mentioning that this is related to the electrocatalytic activity of PPY for the oxidation of methanol [180].

On the other hand, Nafion/PANI composites seem to be the choice for mixed conduction in the catalyst layer of PEMFCs due to their high electronic and protonic conductivities [181,182]. In the very first reports, PANI is used as an electron-conducting matrix, where microparticles of the catalyst can be embedded and protected against particle loss and contamination from reactants [183]. The electrocatalytic activity of Pt microparticles, which were electrodeposited in PANI, showed excellent long-term stability for the electro-oxidation of methanol in acidic solutions [183]. PANI can be easily synthesized by electropolymerization of aniline with very good reproducibility and is stable in a wide potential window. PANI exists in four redox states, which are the leucoemeraldine base (reduced form), emeraldine base (half-oxidized form—EB), emeraldine salt (half-oxidized protonated form—ES) and pernigraniline base (oxidized form) [184]. Among these, only the half-oxidized form, ES, shows high conductivity, while the other forms show either low conductivity or act as dielectrics [185]. In addition, PANI exhibits high permeability to O_2_ and is stable in oxygenated acidic solution. Coutanceau et al. and Croissant et al. have investigated the electrocatalytic activity of different mass loadings of Pt particles in PANI for both the oxidation of H_2_ and the reduction of dioxygen for use in PEMFCs [186,187]. In their pioneering work, the authors concluded that the lowest Pt loading in a PEMFC for both negatrode and positrode electrodes equals to 0.35 mg/cm^2^, which translates to 3 kW per gram of platinum. Such values are also realistic for PEMWEs as indicated by our extensive review on PEM water electrolyzers [188].

Lai et al. investigated two routes of depositing Pt particles in the matrix of a Nafion/PANI composite based on electrochemical and chemical reduction of K_2_PtCl_6_ [165]. With regards to the electrochemical reduction, although it showed a good dispersion of the Pt particles in the Nafion/PANI composite, the performance of the electrode showed inferior activity compared to the formation of Pt by chemical reduction. This was assigned to the nature of Pt growth in the polymer matrix. The stability of the electrode containing Pt particles grown with the chemical method for the ORR (positrode) was assessed over continuous cycling between 0.0 and +0.6 V at 5 mV/s in 0.5 M H_2_SO_4_. The electrode showed a 60% degradation over the first 300 h of operation, but then it stabilized. The authors acknowledge the importance of testing such an electrode under realistic PEM operation, since the oxygen flux is very different, and moreover, the stability of the polymer cannot be judged and it is not directly comparable to the RDE conditions. Barthet et al. prepared Nafion-doped PANI composites by two chemical methods, which differ essentially in the timing of the doping step [189]. The most effective method in terms of preparation of a polymer-based electrode for electrochemical processes, such as PEMFCs, was when polyemeraldine base dissolved in NMP was directly doped with acid-exchanged Nafion. The homogeneous composite polymer showed an electronic conductivity of 0.3 S/cm and an apparent diffusion coefficient for proton and Li ions between 10^−6^ and 10^−8^ cm^2^/s. These values can be compared to the Li diffusion coefficients found for electrochemically synthesized Nafion/PPY composites [189].

**Figure 22:**
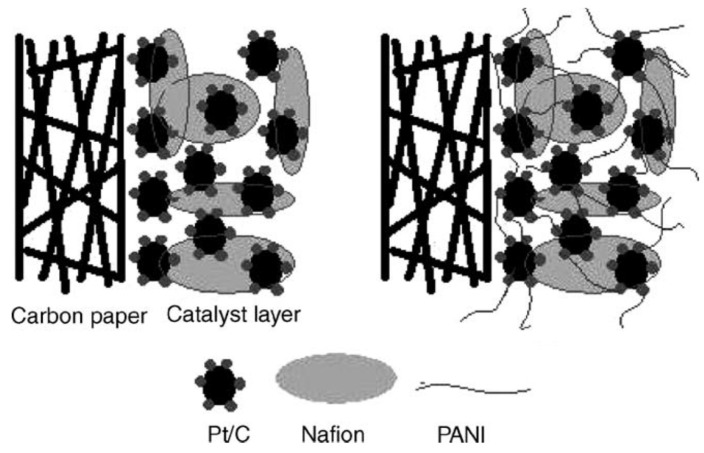
Schematic representation of the role of PANI nanofibers in connecting the catalyst particles embedded in the Nafion matrix. Reprinted with permission from Ref. [191]. Copyright 2006 Elsevier.

Gharibi et al. synthesized PANI nanofibers by electropolymerization of aniline and trifluoromethanesulfonic acid on a Nafion-containing catalyst layer of a gas diffusion electrode (GDE) [190]. The introduction of PANI in the Nafion-containing catalyst (Pt) layer reduced the polarization resistance by minimizing the ohmic resistance, as well as the charge transfer and mass transport limitations. Moreover, the Pt utilization for the ORR was increased almost 30%, a fact that can eventually reduce the Pt amount [191]. A schematic diagram where the PANI nanofibers form a conducting network among the Pt particles is given in Figure 22. It should be noted though that the GDE was not tested in a PEMFC and the characterization was done as a half cell.

An interesting work, in which PANI did not induce electronic conductivity when mixed with Nafion, was presented by Yang et al. [192]. The reason for this behaviour was not investigated, but the authors did not experience any short-circuit when the membrane was mounted in a single cell PEMFC. A possible reason could be the fact that the amount of PANI did not reach the percolating threshold for electronic conduction. The composite membrane was synthesized by an in-situ chemical polymerization technique, where a pre-cleaned and pre-treated Nafion membrane was immersed in an aniline solution that was mixed with (NH_4_)_2_S_2_O_8_. The authors showed that the composite membrane had a superior performance compared to Nafion 112 at 60% RH at 30 °C. The conductivity was approx. 30 mS/cm, whilst for the Nafion 112, it was approx. 18 mS/cm. More interestingly, the composite membranes had lower water uptake capacity, and the improved proton conductivity was hypothesized to be due to the conjugated bonds in PANI. As expected, the single fuel cell testing showed a better performance with the composite membranes when operated with dry gas feed. On the other hand, Berezina et al. prepared composite membranes based on PANI and a Nafion-type (MF-4SC) membrane by a chemical template synthetic method [193]. The electronic conductivity of PANI alone was found in the range of 10^−2^ to 10^3^ S/m, while the composite PANI/MF-4SC had a total conductivity (electronic and protonic) of the order of 1.2 to 5.5 S/cm. The ion exchange membrane alone had a protonic conductivity of the range of 1 to 14 S/m. It can be seen that a synergistic effect in the conductivity values of the composite membrane did not occur and multiple factors may be responsible for this behaviour, such as morphological parameters and the redox inhomogeneity of the composite polymer.

Wolz et al. used the spray coating method to develop a layer-by-layer assembled electrode [194]. Pt nanoparticles were synthesized by the polyol method on PANI films and on single walled carbon nanotubes (SWCNTs). The multilayered electrode was applied on a Nafion membrane and its presence in the electrode was not necessary. This architecture improved the Pt utilization by a factor of three, yielding a performance of 2.7 W/mg_Pt_.

One of the first studies where a Nafion/PANI composite was employed as a positrode for the ORR in a PEMFC was presented by Kakaei [195]. The cathode was prepared by mixing PANI, which was doped with trifluoromethane sulfonic acid, with Vulcan XC72 carbon. Then the mixture was impregnated with Pt particles by adding H_2_PtCl_6_, which was reduced by NaBH_4_ solution. This electrode was compared with a standard Pt/C electrocatalyst in Nafion and the performance of the PANI modified electrode was improved by 1.82 times. PANI was in the form of fibres, which formed an electron-conducting network along with the Vulcan XC72 carbon, improving the performance of the Pt particles due to improved electrical conduct of the particles.

Among other conducting polymers, poly(3,4-ethylenedioxythiophene) (PEDOT) has attracted interest due to its high electronic conductivity and optical transparency [196,197,198]. In combination with sulfonated poly(2,6-dimethyl 1,4-phenylene oxide) (sPPO), mixed conducting membranes can be prepared. Liu et al. developed a highly transparent, mixed conducting polymer composite of PEDOT:sPPO [199]. The synthesis was performed by mixing an aqueous solution of sPPO with EDOT and Fe(SO_4_)_3_ 9H_2_O, initiating the polymerization reaction. The ratio between PEDOT and sPPO was adjusted by the ratio of sPPO and EDOT. After DMSO treatment, an unprecedented enhancement of the electronic conductivity was observed, that reached as high as 10 S/cm, without compromising the proton conductivity. The latter reached up to 20 mS/cm and the increase in the electronic conductivity was attributed to the chain rearrangement and the improvement of the connectivity between the conducting grains of PEDOT. McFarlane et al. prepared Nafion/PEDOT:PSS mixed conducting membranes by simply mixing Nafion and PEDOT:PSS solutions and then drop-casting the dispersions on glass substrates [200]. The membranes were annealed in a vacuum oven, resulting in freestanding, semi-transparent (depending on the amount of PEDOT:PSS), water-insoluble and mechanically-robust membranes. The electronic conductivity was measured in ambient conditions with a four-probe set up, while the protonic in 4 M H_2_SO_4_ was measured in a special glass cell. Composite membranes containing 12% PEDOT:PSS exhibited an electronic conductivity of approx. 7 mS/cm and the ionic was of the order of 103 mS/cm, which is the same as a fully wetted Nafion at 80 °C and 100 RH. Although the purpose of these membranes is their use in systems of artificial photosynthesis for solar water splitting and hydrogen production, it will be very interesting to incorporate into PEMFCs and PEMWEs electrodes.

A few very interesting mixed electron proton-conducting membranes and standalone materials have been recently developed, but unfortunately have not been tested in PEMFCs or PEMWEs. They are worth presenting though for their innovation character as well as their possible applications beyond PEMFCs and PEMWEs, as in artificial photosynthesis, sensors and energy storage devices. In parallel though, we aim to inform and inspire the reader for potential use as electrodes in PEMFCs and PEMWEs.

**Figure 23:**
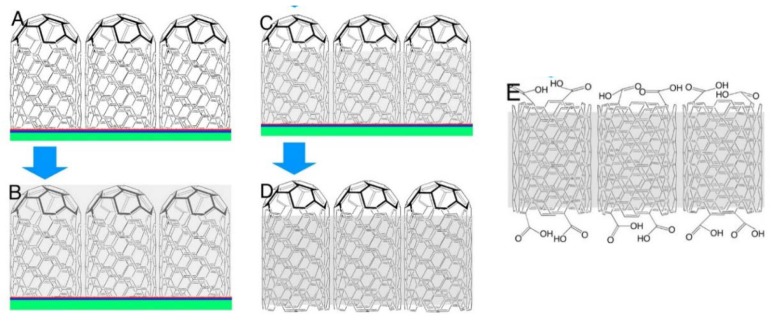
VANT membrane synthesis route. In green is the Si wafer, in blue an alumina support and in red an iron catalyst where the CNTs were grown (**A**); in grey the epoxy coating (**B**); tip exposure (**C**); removal of wafer and catalyst (**D**); tube opening via O_3_ treatment (**E**). Reprinted with permission from Ref. [203]. Copyright 2014 American Chemical Society.

Ijeri et al. combined Nafion with multi-walled carbon nanotubes (MWCNTs) beyond their percolation threshold with a very simple mix-and-cast method [201]. The authors tested the Nafion/MWCNTs under dry and wet conditions in ambient temperatures and found that the electronic conductivity increased with increasing MWCNTs content and reached approx. 0.37 mS/cm in dry conditions. After wetting, the electronic conductivity decreased by approx. 30% but as expected the protonic conductivity increased. The protonic conductivity reached the 5 mS/cm in the presence of 5% wt. of MWCNTs. In a follow-up work of the same group, the authors prepared the same type of composite membranes with the difference that the MWCNTs were now aligned forming distinct electron conduction paths [202]. The MWCNTs were grown with chemical vapor deposition (CVD) on Si substrates, which were patterned by optical lithography. The MWCNTs on Si were then flooded with Nafion solution and after evaporation the membrane was detached by immersion in HF. In this case, the electronic conductivity in dry conditions was improved to 0.57 mS/cm as opposed to 0.37 mS/cm in their previous work. The protonic conductivity was also improved from 5 to 9 mS/cm. Pilgrim et al. developed mixed conducting membranes made of vertically-aligned carbon nanotubes (VANT) [203]. The synthesis of the membrane was conducted through three basic steps: CNTs growth, epoxy coating and CNTs exposure (Figure 23).

**Figure 24:**
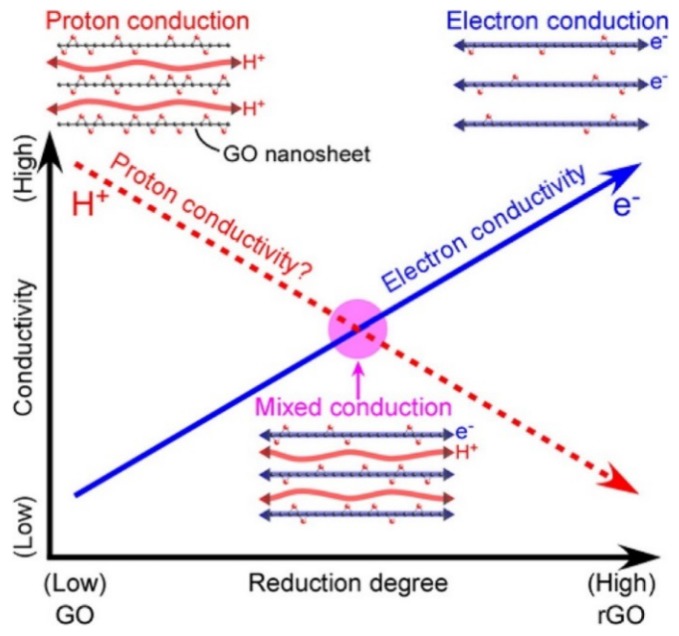
The concept behind the degree of GO reduction, which increases the electronic conductivity but decreases the protonic one. Reprinted with permission from Ref. [204]. Copyright 2014 American Chemical Society.

The electrical transport was ohmic with a conductivity of 495 mS/cm. After wetting the membrane, the proton conductivity was enabled through the bore of the CNTs and the authors hypothesize that the transport was due to the Grotthuss mechanism. The proton conductivity was measured by a peculiar set up in, where the proton transport was monitored by the absorption spectrum of bromophenol blue. The proton conductivity of the VANT membrane was found to be half of Nafion’s, which was measured in the same experimental set up. The work lacks evidence on the transport mechanism and also conductivity dependencies over varying water levels and temperatures, parameters that will be very interesting to further investigate. Another single component, carbon-based mixed conductor was shown by Hatakeyama et al. [204]. In this work, the degree of GO reduction by photo and thermal methods can tune the mixed conductivity of the GO/rGO membrane. This is schematically illustrated in Figure 24.

The protonic conductivity in this type of single component mixed conductor is attributed to the epoxide groups in between the GO sheets that facilitate the proton transport [205]. The authors found that the material with the optimum degree of reduction had the same electronic and protonic conductivity of approx. 10^−4^ S/cm at 90% RH at room temperature. In a follow up work of the same group, the rGO was modified with sulfate ions (r-sGO); the protonic conductivity at 90% RH in room temperature was increased to 3 × 10^−2^ S/cm and the electronic to 2 × 10^−2^ S/cm [206]. Finally, another single component mixed conductor is based on hierarchical nanostructured WO_3_ [207]. The bioinspired hexagonal WO_3_ nanorods (h-WO_3_) were synthesized by the hydrothermal method. The proton conductivity of this material relies on the water content of the hydrous WO_3_ (h–WO_3_·*n*H_2_O), which was determined by thermogravimetric analysis. On the other hand and in order to induce electronic conductivity, the h–WO_3_·*n*H_2_O was reduced after annealing in reducing atmosphere (5% H_2_ in N_2_). At room temperature, the electronic conductivity was approx. 0.6 S/cm and decreased with increasing temperature, implying a metallic-like behaviour. The protonic conductivity reached the 1 mS/cm at room temperature at 50% RH, but it increased to 2.7 and 3.7 mS/cm at 60 and 90 °C, respectively. The material was assessed as a capacitor and showed good capacitance with fast charge/discharge capability and very good stability. It would be interesting to see if it can be employed as a mixed conducting component surface modified with Pt nanoparticles in the positrode of a PEMFC.

## 6. Summary, Challenges, Perspectives and Future Directions

In this review, we started with a brief account of the recent status, as well as the targets set for PEM-based energy systems in order to compete with the current energy technologies. There is surely a requirement and trend of reduced cost of PEM systems, but it looks like the rate is not as significant during the last few years as a decade or more ago. One of the main reasons is the cost of certain components, such as the PEM (e.g., Nafion) and the electrocatalysts (noble metals). We have recently reviewed the latter and it seems that PEM systems have a long way to go to escape from the use of noble metals as electrocatalysts [188]. There is an enormous amount of research on earth-abundant electrocatalysts, but the efforts to actually utilise them in operating PEM systems are few. This is definitely one of the main barriers the PEM world should realise and improve in order to further reduce the costs.

When it comes to the PEM, our review indicates that the “conductivity gap” still exists between the upper temperature limit for Nafion-based membranes and the lower temperatures of sufficient conductivity for the non-fluorinated systems based on acid-doped PBI and SPEEK. Apparently, metal oxide fillers do not significantly improve the proton conductivity at temperatures higher than 80 °C and low RH levels for PFSA polymers. At higher temperatures, when water evaporates from the polymer, the channels start to contract, breaking at the same time the connections among the filler particles. How can we then make percolating networks of the particles of the filler? Should such a self-standing network be synthesized first and then grow or infiltrate the polymer around it? On the other hand, the metal oxide fillers seem in some cases beneficial for temperatures below 80 °C, but are the results reproducible and is there a real gain considering the cost of that extra component, as well as the disadvantages of the increased brittleness and the of LT-PEMFC operation (catalyst poisoning, flooding etc.)? We have found a few promising materials, where Nafion is mixed with MOFs as well as graphene oxide (GO) that show quite high conductivity values even at 120 °C and low RH [61,68,80]. The main issue is that these membranes were not tested in full PEMFCs; their stability is not assessed and it could be that the costs of GO and the MOFs turn out to be prohibitive.

The acid-doped PBI-based membranes show very good conductivities at high temperature and low relative humidity, as well as good power densities, but the majority of the systems operate well above 150 °C and there are no reports at lower operating temperatures close to the US DOE target of 120 °C. Zirconium hydrogen phosphate has been shown to yield significant conductivity up to 200 °C, but the detailed interaction mechanism between this filler and the PBI matrix is not clear, and the stability with this composite membrane at such high temperature has hardly been studied. One must suspect that this like many other phosphates more than anything else acts as a source of phosphoric acid upon decomposition at high temperature. Composite membranes containing heteropolyacids (HPA) exhibit high proton conductivity under anhydrous conditions at 150 °C attributed to HPAs providing additional surface functional sites through the composite membrane to promote the proton transport because protons are transferred on the surface of the HPA [208]. A balance between proton conductivity and mechanical strength of the membrane may be required for a promising PBI-based composite membrane. Recently, PEM with co-doped MOFs have demonstrated significant enhancement in proton conductivity, which is attributed to the synergistic effect between the two fillers leading to ionic channels with better connectivity. However, these composites have not found any practical applications in fuel cells yet. A further challenge is to make an electrocatalytic layer that would be compatible to both membrane and catalyst.

SPEEK is a cheaper alternative to Nafion, but its conductivity is in general lower. Moreover, the high degree of sulfonation leads to poor mechanical stability. As a consequence, inorganic fillers are, as we have seen, tried as remedy. Metal oxide composite membranes based on SPEEK show again lower conductivities than the analogues in Nafion, but certain composites with MOFs show improved conductivities [58,63,153]. Apparently, MOFs, as well as cavity-modified ones with acidic groups, improve the proton conduction, an effect observed also in the Nafion case. Again, full PEMFCs tests are missing and they are of paramount importance in order to assess the compatibility, stability and lifetime of such composite membranes. Another factor is also the cost of these structures, if proven to be successful. A technoeconomic analysis on the viability of MOFs-doped PEM should be conducted.

An interesting composite based on polysilsesquioxane was presented by Pezzin et al. [157]. This composite showed a proton conductivity of 142 mS/cm at 120 °C and 100% RH, but the performance and the stability of the composite membrane was not tested in a full PEMFC. Such hybrid membranes with Si–O networks look promising and may assist in the formation of percolating networks in the parent membrane during low RH conditions and temperatures around 120 °C. A similar study was conducted by Nam et al. where they also observed high proton conductivity of 157 mS/cm at 120 °C and 100% RH from a Nafion/sulfonated poly(phenylsilsesquioxaine) nanocomposite membrane, which is higher than that of Nafion [46]. A few follow-up works worth pursuing have been found [209,210,211].

We have introduced the definition of the negatrode and positrode electrodes, as used in proton ceramic fuel cells and electrolysers, and we are among the few to review developments on mixed electron proton-conducting polymer materials for use in the CL. Regarding the progress in mixed conducting polymers, we see a lack of measurements in full PEMFCs. Some of the reports are even performing the conductivity measurements in liquid electrolytes and in three-electrode configurations. This is useful as it is easier to set up such measurements, rather than full PEMFCs, and they can give immediate indications for the efficiency and performance as means of fast materials screening. On the other hand, the operational conditions are quite different, in terms of both temperature, but also concentration and mass transport of the reactants and products towards and from the active sites, respectively. The latter can also have a big impact on the stability and lifetime of the mixed conductor. Another important aspect that is missing in the literature is a more complete physicochemical and electrochemical characterization on different RH levels and temperatures, especially at elevated ones (above 100 °C). This is particularly important, as there are no synchronized efforts in the research for high temperature-tolerant mixed conductors that could follow up the developments on the high temperature membranes.

PEDOT is also a promising electronically-conducting polymer beyond PANI and it should be explored more, especially in full PEMFCs and also in terms of stability at high operating temperatures. The unprecedented increase in its electronic conduction after treatment with DMSO as presented by Liu et al. [199], should be studied further. The fundamental understanding behind this effect could lead to an increase in performance in other electron-conducting polymers and also to the discovery of other polymer systems that can turn into electron conductors.

The development of single-component mixed conductors based on oxides is an important approach, as oxides can tolerate high operating temperatures; however, certain aspects must be taken into account. For example, how is the electrocatalyst deposited and how efficient is the mixed conductor/electrocatalyst interface? It is important to investigate how the ions and electrons are transported and react on that interface. What is the impact of the morphology of the mixed conductor (e.g., tubes, rods, nanoparticles etc.) and its compatibility with the PEM?

At the very end, we would like to look back at the skepticism we expressed at the outset as to the effects of fillers of various kinds introduced to proton-conductive polymers. We have indeed seen some cases of remarkable improvements in conductivity, power density, stability and operation at higher temperatures and lower RH. However, the remarkable majority of them report improvements within limits that may well be statistical variation—a variation or reproducibility usually not reported. We may suspect underreporting of negative effects of fillers. The matrix of acid doping and fillers and their interactions is often not completely mapped. We have seen many cases of large improvements of protonic conductivity accompanied by a remarkable drop in activation energy to levels only associated with liquid phase transport, shedding doubt on the characterisation of the supposedly solid-state material. We have seen few credible rationalisations of why fillers work. For example, some refer to the use of particles of proton-conductive materials, all of which are recently understood to exhibit only protonic conduction in adsorbed water. This leads us to encourage that studies of PEM composites onwards emphasise well-characterised microstructures (bulk, grain boundaries, phase boundaries, internal surfaces) and a well-founded assignment of protonic conduction appropriate to bulk solid polymer and ceramic phases and liquid phases, adsorbed water layers (chemisorbed, physisorbed), and interfaces (e.g., through space charge effects).

## Figures and Tables

**Table 1:** US DOE technical targets for PEMs for transportation applications, adapted from Refs. [11,12,13].

Characteristics	2013 Status	2020 Targets
Maximum oxygen/hydrogen crossover *	<1.8 mA/cm^2^	2 mA/cm^2^
Maximum operating temperature	120 °C	120 °C
Membrane conductivity	0.002–0.04 S/cm	0.1 S/cm (120 °C)
		0.07 S/cm (Ambient)
		0.01 S/cm (−20 °C)
Area specific proton resistance at maximum	40 kPa: 0.023 Ω cm^2^	0.02 Ω cm^2^
Operating temperature and water partial pressures from 40 to 80 kPa	80 kPa: 0.012 Ω cm^2^	
Minimum electronic area specific resistance		1000 Ω cm^2^
Cost	$18/m^2^	$20/m^2^
Durability (mechanical & chemical)	>20,000 cycles w/<10 sccm crossover, >2300 h	20,000 cycles w/<10 sccm crossover, 500 h

* Tested in MEA at 1 atm O_2_ or H_2_ at nominal stack operating temperature, humidified.

**Table 2:** Comparison of proton conductivities of Nafion-based composite membranes and their PEMFC performance.

Membrane	Conductivity/Activation Energy	Temperature (°C)	RH (%)	Maximum Power Density in PEMFC	Ref.
Nafion/12 wt.%Phytic@MIL101	228 mS/cm and 15.14 kJ/mol	80	100	Not reported	[54]
Nafion/10 wt.% SAFHSS	100 mS/cm	100	100	Not reported	[45]
Nafion/4 wt.% GO	170 mS/cm and 12.98 kJ/mol	80	100	100 °C and RH = 25% 212 mW/cm^2^	[76]
Nafion/0.05 wt.%s-SWCNTs	15.5 mS/cm	100	100	65 °C 650 mW/cm^2^	[77]
Nafion/5 wt.% sPPSQ	157 mS/cm	120	100	Not reported	[46]
Recast Nafion/20 wt.% ZrSPP	50 mS/cm	110	98	100 °C 700 mA/cm^2^ @0.4 V *	[78]
Nafion/15 wt.% Analcime	437.3 mS/cm	80	100	Not reported	[79]
Nafion/1 wt.% ZIF-8@GO	280 mS/cm and 13.2 kJ/mol	120	40	Not reported	[66]
Nafion-0.6/UiO-66-NH_2_ + UiO-66-SO_3_H	256 mS/cm	90	95	Not reported	[80]
Nafion-0.6/GO@UiO-66-NH_2_	303 mS/cm	90	95	Not reported	[68]
Nafion/3 wt.% CPO-27(Mg)	11 mS/cm	50	99.9	50 °C and RH = 100%	[67]
				818 mW/cm^2^	
				80 °C and RH = 100%	
				591 mW/cm^2^	
Nafion/1 wt.% SZM	2.96 mS/cm	80	35	80 °C and RH = 35%	[61]
				550 mA/cm^2^ @0.3 V	
Nafion/1 wt.% F-GO	17 mS/cm	70	20	70 °C and RH = 20%	[81]
				300 mW/cm^2^	
Nafion/3 wt.% Fe_3_O_4_-SGO	11.62 mS/cm and 21.41 kJ/mol	120	20	120 °C and RH = 25% 258.82 mW/cm^2^	[82]
Recast Nafion/14.3 wt.% SiO_2_-PWA	26.7 mS/cm and 11.2 kJ/mol	110	70	110 °C and RH = 70%	[70]
				540 mA/cm^2^ @0.4 V *	
meso-Nafion/ 19 wt.% H_3_PW_12_O_40_	72 mS/cm	80	40	80 °C and RH = 50%	[83]
				541 mW/cm^2^	

* Power density not reported, but we give the current density at the respective applied voltage.

**Table 3:** Comparison of proton conductivities of H_3_PO_4_–PBI-based composite membranes and their PEMFC performance.

Membrane	Acid Doping Level (mol H_3_PO_4_ per r.u. PBI)	Conductivity/Temp/%RH/	Maximum Power Density in PEMFC	Ref.
PBI/2 wt.% TiO_2_	15.3	130 mS/cm at 150 °C and 10% RH	800 mW/cm^2^@150 °C	[115]
PBI/2 wt.% TiO_2_	Not provided	43 mS/cm at 150 °C	450 mW/cm2@175 °C	[116]
PBI/5 wt.% SiO_2_	10	102.7 mS/cm at 180 °C and 0% RH	240 mW/cm^2^@165 °C	[110]
PBI/5 wt.% SiO_2_	13.4	113 mS/cm at 180 °C and 0% RH	Not reported	[119]
		23.8 kJ/mol		
PBI/5 wt.% ZrP	15.4	200 mS/cm at 180 °C and 0% RH	Not reported	[119]
		19.4 kJ/mol		
PBI/ 15 wt.% ZrP	5.6	96 mS/cm at 200 °C and 5% RH	Not reported	[93]
		16.65 kJ/mol		
OPBI/15 wt.% LAMS	23.4	181 mS/cm at 160 °C and 0% RH	Not reported	[143]
		16.65 kJ/mol		
PBI/4 wt.% Fe_2_TiO_5_	12	78 mS/cm at 180 °C and 0% RH	430 mW/cm^2^@180 °C	[108]
PBI/10 wt.% SNP-PBI	Acid uptake 385 ± 15%	50 mS/cm at 160 °C and 0% RH	650 mW/cm^2^@150 °C	[112]
PBI/Cs_2.5_H_0.5_PMo_12_O_40_	120%	150 mS/cm at 150 °C and 8.4% RH	700 mW/cm^2^@150 °C	[128]
PBI/30 wt.% CsPOMo	4.5	120 mS/cm at 150 °C and 0% RH	600 mW/cm^2^@150 °C	[129]
		6 kJ/mol		
PBI/PTFE	175%	120 mS/cm at 180 °C and 8.5% RH	600 mW/cm^2^@150 °C	[144]
	300%	320 mS/cm at 180 °C and 8.4% RH		
PBI/15 wt.% laponite clay-N	8.2	120 mS/cm at 150 °C and 12% RH	220 mW/cm^2^@150 °C	[141]
OPBI/20 wt.% AMS	31.25	125 mS/cm at 160 °C and 0% RH	Not reported	[145]
		16.15 kJ/mol		
Py-PBI/1.5 wt.% PGO	9.93	76.5 mS/cm at 140 °C and 0% RH	360 mW/cm^2^@120 °C	[136]
		18 kJ/mol		
PBI/2 wt.% GO	13	129.7 mS/cm at 165 °C and 0% RH	378 mW/cm^2^@165 °C	[134]
		24.7 kJ/mol		
PBI/5 wt.% ZIF-8+ZIF-67	Acid uptake 157%	91 mS/cm at 200 °C and 0% RH	Not reported	[57]
		19.6 kJ/mol		
OPBI/7 wt.% Okao	24.746	72 mS/cm at 160 °C	Not reported	[142]
		8.75 kJ/mol		
OPBI/7 wt.% OMMT	25.479	92 mS/cm at 160 °C	Not reported	[142]
		8.17 kJ/mol		
PBI/1 wt.% p-MWCNTs	12.4	110 mS/cm at 160 °C and 0% RH	780 mW/cm^2^@140 °C	[132]
		25.1 kJ/mol		
PBI/10 wt.% nanoCaTiO_3_	127.2%	28 mS/cm at 160 °C and 0% RH	570 mW/cm^2^@160 °C	[146]
		21.32 kJ/mol		
